# Structural Modification and Conjugation Strategies of Antimicrobial Peptides for Topical Anti-Infective Applications

**DOI:** 10.3390/antibiotics15040390

**Published:** 2026-04-10

**Authors:** Edson Reinaldo Júnior, Sabrina Fantini Do Nascimento, Janaína Teixeira Costa De Pontes, Keren Yuki Takada, Vanderson De Jesus Silva, Fernando Rogério Pavan, Cesar Augusto Roque-Borda

**Affiliations:** 1 Tuberculosis Research Laboratory, Department of Biological Sciences, School of Pharmaceutical Sciences, Universidade Estadual Paulista (UNESP), Araraquara 14800-901, São Paulo, Brazilj.pontes@unesp.br (J.T.C.D.P.);; 2 Departament of Biological and Helth Sciences, University of Araraquara (UNIARA), Araraquara 14801-340, São Paulo, Brazil; kytakada@uniara.edu.br; 3 Vicerrectorado de Investigación, Universidad Católica de Santa María, Arequipa 04001, Peru

**Keywords:** antimicrobial peptides, bioinspired antimicrobials, peptide conjugation, topical delivery

## Abstract

Antimicrobial peptides (AMPs) have re-emerged as promising anti-infective agents, particularly against multidrug-resistant bacteria; however, their therapeutic development remains constrained by proteolytic degradation, host cell toxicity, and rapid systemic clearance. Rather than focusing solely on sequence discovery, recent efforts have shifted toward structural and supramolecular modification strategies aimed at improving stability, selectivity, and pharmacological performance. This review critically analyzes intramolecular modifications—including phosphorylation, glycosylation, acetylation, methylation, and backbone cyclization—that modulate peptide conformation and resistance to enzymatic degradation. In parallel, extramolecular approaches such as PEGylation, lipidation, and conjugation to antibiotics, siderophores, or antibodies are examined in the context of enhanced targeting and prolonged bioavailability. Particular emphasis is placed on localized delivery systems, including hydrogels, polymeric films, and nanofibrous scaffolds, which enable spatially controlled administration and mitigate systemic exposure. By integrating evidence from ex vivo and in vivo infection models, this work delineates the translational potential and remaining bottlenecks of chemically engineered AMP platforms for skin and soft tissue infections.

## 1. Introduction

Antimicrobial resistance (AMR) is progressively reshaping the therapeutic landscape of infectious diseases, eroding the reliability of antibiotics that have underpinned clinical practice for decades [1]. Skin, soft tissue, and chronic wound infections exemplify this challenge, as both Gram-positive and Gram-negative pathogens increasingly display multidrug-resistant phenotypes that compromise empirical and targeted therapies alike [2]. Although antimicrobial stewardship and surveillance remain essential, the limited influx of truly novel antibiotic classes and the high failure rate in late-stage clinical development have exposed structural weaknesses in the current drug discovery paradigm [3]. These constraints have intensified the search for antimicrobial modalities that operate beyond the classical small-molecule framework.

Within this context, antimicrobial peptides (AMPs) represent a mechanistically distinct class of anti-infective agents [4,5,6]. As integral components of innate immunity across diverse organisms, AMPs combine cationic charge and amphipathic organization to interact dynamically with microbial membranes and intracellular targets. Their antibacterial activity extends beyond membrane permeabilization, encompassing interference with metabolic pathways, modulation of stress responses, and, in certain cases, immunoregulatory signaling [7,8]. Importantly, although AMPs frequently engage fundamental physicochemical features of bacterial cells—potentially complicating resistance acquisition—adaptive mechanisms such as surface remodeling, efflux regulation, and proteolytic degradation demonstrate that resistance remains biologically feasible. Consequently, therapeutic optimism must be tempered by a realistic appraisal of these evolutionary pressures.

Despite their mechanistic versatility, native AMPs rarely fulfill the pharmacological requirements necessary for systemic administration. Rapid enzymatic degradation, dose-dependent cytotoxicity, limited tissue penetration, and unfavorable pharmacokinetic behavior continue to restrict clinical applicability [9,10]. Rather than abandoning the peptide scaffold, contemporary research has increasingly reframed these liabilities as engineering challenges. Chemical and structural modifications have therefore emerged as central strategies to recalibrate peptide stability, selectivity, and biodistribution.

Intramolecular modifications—including phosphorylation, glycosylation, acetylation, methylation, and backbone cyclization—enable controlled manipulation of conformation, charge distribution, and protease susceptibility. Complementarily, extramolecular strategies such as PEGylation, lipid conjugation, and hybrid constructs with antibiotics, siderophores, or antibodies extend functional versatility by enhancing targeting capacity, modulating membrane interactions, and prolonging bioavailability [11,12,13,14]. These approaches have generated a spectrum of architecturally diverse peptide platforms that blur the boundaries between classical antibiotics, biologics, and biomaterials.

Parallel to molecular optimization, advances in formulation science have significantly influenced the translational trajectory of AMPs, particularly in localized infections where high local concentrations can be achieved without systemic toxicity [15,16,17]. Hydrogel matrices, polymeric films, nanofibrous scaffolds, and particulate delivery systems provide physical protection against degradation while enabling spatially controlled release. When evaluated using ex vivo skin models and in vivo infection systems, such formulations offer a more clinically relevant assessment of therapeutic performance than reductionist in vitro assays alone. The convergence of peptide engineering and advanced delivery technologies suggests that the future of AMP therapeutics lies not in mimicking conventional antibiotics, but in strategically integrating chemical design with context-specific administration.

## 2. Mechanisms of Action of Antimicrobial Peptides Against Bacterial Pathogens

### 2.1. Membrane Disruption

The progressive erosion of antibiotic efficacy has redirected attention toward bacterial structures that are indispensable and difficult to bypass through mutational adaptation. Among these, the cytoplasmic membrane constitutes a functionally essential and bioenergetically vulnerable target. Unlike enzyme-specific antibiotics, membrane-active agents disrupt physicochemical integrity, ion gradients, and metabolic homeostasis, and may therefore retain activity against metabolically quiescent or slow-growing bacteria. Moreover, membrane targeting can partially circumvent permeability limitations that restrict intracellular drug accumulation [18]. Despite these advantages, therapeutic application demands strict selectivity. Because lipid bilayers are conserved across biological systems, insufficient discrimination between bacterial and mammalian membranes may translate into hemolysis or host cytotoxicity. This requirement largely explains the limited number of clinically approved antibiotics whose primary mechanism relies on membrane disruption [19].

In Gram-positive bacteria, the cell envelope consists of a thick peptidoglycan matrix formed by alternating *N*-acetylglucosamine (NAG) and *N*-acetylmuramic acid (NAM) residues crosslinked by peptide bridges. Teichoic and lipoteichoic acids embedded within this structure confer a net negative surface charge, contribute to metal ion homeostasis, and participate in host–pathogen interactions [20]. The absence of an outer membrane renders the cytoplasmic membrane and associated precursors more accessible than in Gram-negative organisms, where an additional permeability barrier modulates AMP entry [20,21]. AMP-mediated membrane disruption is mechanistically diverse. Some peptides destabilize lipid bilayers through electrostatic attraction followed by insertion and pore formation. Others interfere with cell wall biosynthesis without immediate membrane rupture. Peptides targeting peptidoglycan assembly frequently act by sequestering essential intermediates rather than directly inhibiting enzymatic activity, thereby indirectly compromising structural integrity [22,23].

Lantibiotics exemplify this dual mechanism. Class A lantibiotics such as nisin bind lipid II, a central precursor in peptidoglycan synthesis, simultaneously blocking cell wall assembly and promoting pore formation [23,24,25]. Although lipid II is conserved in both Gram-positive and Gram-negative bacteria, its accessibility differs substantially due to outer membrane shielding in Gram-negative species. Structural exposure, therefore, becomes a determinant of susceptibility rather than target absence. Alamethicin, an amphipathic α-helical peptide derived from *Trichoderma viride*, demonstrates a concentration-dependent transition from surface alignment to transmembrane insertion, forming barrel-stave pores. In this configuration, hydrophobic residues interact with the lipid core while hydrophilic domains line the pore lumen. Although toroidal and carpet-like models have been described for other AMPs, pore formation remains the predominant mechanism attributed to alamethicin in Gram-positive systems [26].

Not all membrane-associated effects culminate in immediate lysis. Vitellogenin, a yolk precursor protein in oviparous organisms, exerts bactericidal activity against *Staphylococcus aureus* through selective binding to lipoteichoic acid. Its inactivity against protoplasts and neutralization upon preincubation with LTA support a mechanism dependent on structural engagement rather than membrane perforation [27]. These findings underscore that membrane targeting encompasses a spectrum of interactions ranging from energetic collapse to precursor sequestration and cell wall destabilization.

Although membrane disruption represents one of the most extensively characterized antimicrobial mechanisms of AMPs, bacterial pathogenicity frequently extends beyond planktonic growth. In clinical settings, many infections are sustained by structured microbial communities rather than isolated cells. These multicellular assemblies introduce additional physiological barriers that can significantly alter antimicrobial susceptibility. Consequently, understanding how AMPs interact not only with individual bacterial membranes but also with organized bacterial populations is essential for evaluating their therapeutic potential. In this context, increasing attention has been directed toward the capacity of antimicrobial peptides to interfere with the formation and stability of bacterial biofilms.

### 2.2. Biofilm Inhibition

Biofilms represent a highly coordinated mode of bacterial organization in which cells are embedded within a self-produced extracellular matrix adherent to biotic or abiotic surfaces. This structural arrangement confers collective resilience, enhancing tolerance to immune clearance, oxidative stress, nutrient limitation, and antimicrobial exposure [28]. Importantly, biofilm-associated phenotypes are not static; they are shaped by species composition, metabolic gradients, and environmental pressures, resulting in heterogeneous physiological states within the same community [29]. Biofilm establishment is initiated by surface attachment, a process governed by both nonspecific physicochemical interactions and receptor-mediated adhesion. On inert substrates, hydrophobic forces and electrostatic interactions often predominate, whereas on host tissues, microbial adhesins mediate selective binding to extracellular matrix components. *S. aureus*, for example, expresses surface proteins that recognize collagen and other host-derived ligands, facilitating stable colonization [30]. Following attachment, bacteria transition toward matrix production, secreting extracellular polymeric substances (EPS) composed of exopolysaccharides, structural proteins, and extracellular DNA. This matrix not only anchors cells but also creates diffusion barriers that modulate antimicrobial penetration and generate metabolic microenvironments [31].

As the biofilm matures, quorum sensing networks coordinate gene expression programs associated with stress adaptation, persistence, and communal stability. Ultimately, dispersal events release subsets of cells into the surrounding environment, enabling colonization of secondary sites and contributing to recurrent or chronic infections [31]. Thus, biofilm development should be interpreted as a dynamic, multicellular strategy rather than a passive accumulation of bacteria. AMPs interfere with biofilm biology at multiple levels. Beyond direct bactericidal activity, certain peptides perturb membrane potential within biofilm-embedded cells, destabilize matrix architecture, interfere with quorum sensing signaling cascades, or downregulate the genes required for adhesion and structural maintenance [32]. This multimodal interference distinguishes AMPs from many classical antibiotics that primarily target actively dividing planktonic cells.

Segev-Zarko et al. [33] demonstrated that the synthetic peptide WMR, derived from the Trp-Met-Phe (WMF) motif, exerts pronounced activity against *S. aureus* biofilms. WMR displays a dual mechanism: enzymatic or structural degradation of the extracellular matrix combined with membrane depolarization of resident cells. Matrix disruption enhances permeability, facilitating deeper penetration of antimicrobial agents, while membrane destabilization induces energetic collapse. The simultaneous targeting of structural and cellular components enables activity against both sessile and planktonic populations. Similarly, Li et al. [34] reported that IDR-1018, a synthetic cathelicidin-derived peptide, suppresses biofilm formation in Gram-positive bacteria, including *S. aureus*. Notably, IDR-1018 modulates transcriptional programs associated with polysaccharide synthesis and quorum sensing. Downregulation of these pathways compromises biofilm cohesion and maturation, rendering bacterial communities more susceptible to antibiotic therapy and host immune clearance. These findings illustrate that AMP-mediated biofilm inhibition extends beyond membrane disruption, encompassing regulatory interference and structural destabilization.

### 2.3. Modulation of the Local Inflammatory Response

The biological activity of antimicrobial peptides extends beyond direct bacterial killing and includes active participation in the regulation of host immune responses at sites of infection. Rather than functioning solely as microbicidal agents, many AMPs act as endogenous immune modulators capable of shaping inflammatory intensity and cellular recruitment patterns [35]. This dual functionality reflects their evolutionary origin within innate immunity, where antimicrobial defense and immune coordination are tightly interconnected. Host-derived peptides such as α-defensins and cathelicidins, produced by neutrophils and natural killer cells, exemplify this integration of functions. In addition to membrane-disruptive activity, these peptides display chemotactic properties that promote recruitment of monocytes, T cells, and other effector populations essential for early containment of infection [36]. Their capacity to operate in a chemokine-like manner underscores the conceptual overlap between antimicrobial and immunoregulatory signaling.

Synthetic AMPs have been engineered to preserve or enhance these immunomodulatory properties. Certain analogs reduce bacterial burden while simultaneously modulating local cytokine profiles. For instance, attenuation of tumor necrosis factor-α production in epithelial cells exposed to bacterial components has been observed following treatment with specific synthetic peptides [37]. This selective dampening of excessive inflammatory signaling suggests that AMP activity is not inherently pro-inflammatory but context-dependent, potentially limiting collateral tissue damage while maintaining antimicrobial pressure. Beyond modulation of cytokine production, some peptides directly neutralize bacterial virulence-associated molecules. Interaction with Gram-positive lipoproteins has been shown to suppress the release of pro-inflammatory mediators such as IL-8, IL-1α, IL-1β, IL-6, and MCP-1 [38]. Mechanistically, this effect correlates with the inhibition of intracellular signaling cascades including NF-κB and p38 MAPK, central nodes in inflammatory amplification. In parallel, interference with dendritic cell maturation and migration can reduce subsequent T cell activation, thereby constraining excessive adaptive immune escalation without abolishing antimicrobial efficacy [38].

**Figure 1:**
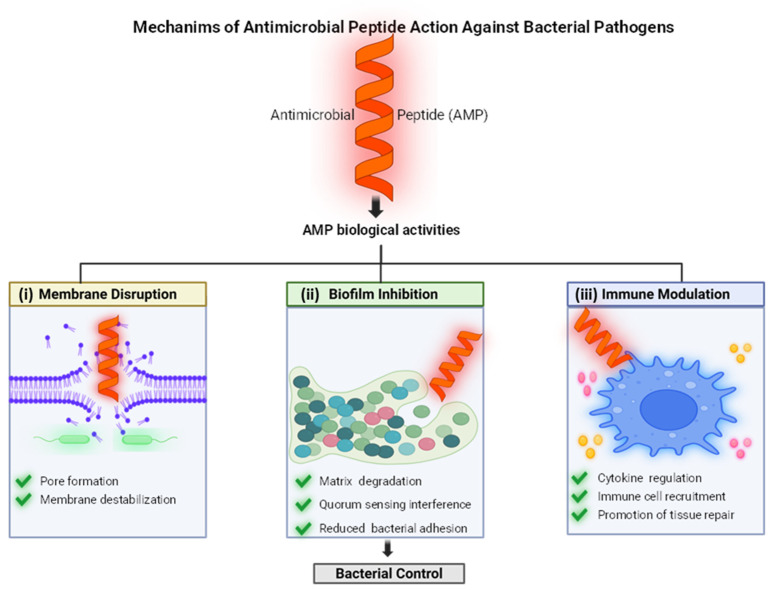
Multifaceted mechanisms of action of antimicrobial peptides. Antimicrobial peptides exert their activity through several complementary biological mechanisms. (i) Membrane disruption, where amphiphilic peptides interact with negatively charged bacterial membranes, leading to pore formation and membrane destabilization. (ii) Biofilm inhibition, involving interference with biofilm matrix integrity, quorum sensing pathways, and bacterial adhesion processes. (iii) Immune modulation, in which peptides regulate cytokine production, promote immune cell recruitment, and contribute to tissue repair processes. The combined action of these mechanisms contributes to effective bacterial control and reduced infection persistence.

Importantly, immunomodulation by AMPs is not restricted to inflammatory suppression. Several peptides contribute to tissue repair dynamics by influencing metalloproteinase activity and epidermal growth factor receptor signaling pathways, enhancing keratinocyte migration and facilitating re-epithelialization during wound healing [38]. Such effects are particularly relevant in infections of the skin and soft tissue, where bacterial clearance and tissue restoration must proceed concurrently. Collectively, these observations indicate that AMP function in Gram-positive infections cannot be reduced to membrane disruption alone (Figure 1). Instead, antimicrobial activity, biofilm interference, inflammatory calibration, and tissue regeneration often occur in parallel, generating a coordinated response that integrates pathogen control with preservation of host tissue integrity.

It is important to note that the mechanisms discussed in this section do not represent an exhaustive classification of antimicrobial peptide activity. Not all AMPs exhibit the same functional profile, and many peptides act through mechanisms that extend beyond the three categories described here. Depending on their structure, physicochemical properties, and biological context, AMPs may interact with intracellular targets, interfere with metabolic pathways, alter membrane-associated signaling, or modulate bacterial stress responses. In addition, several peptides display multifunctional behavior, combining membrane perturbation, antibiofilm activity, and immunomodulatory effects within the same system. For this reason, the mechanisms presented here should be understood as representative examples of the most frequently described modes of action rather than a complete framework encompassing the full diversity of AMP activity.

## 3. AMP Formulations for Topical Applications

### 3.1. Structure and Formation of Topical Systems

The evolution of topical drug delivery platforms reflects a shift from simple vehicles toward structurally engineered systems capable of modulating release dynamics, tissue interaction, and mechanical stability. Contemporary formulations—including hydrogels, semisolid emulsions, polymeric films, and impregnated wound dressings—differ in architecture and fabrication routes but share a unifying objective: the integration of biocompatible matrices with predictable physicochemical behavior suitable for cutaneous application [39]. Crucially, compositional selection and manufacturing strategy are not merely technical considerations; they dictate network organization, diffusion pathways, adhesion properties, and ultimately therapeutic performance [40,41].

#### 3.1.1. Hydrogels

Hydrogels represent one of the most adaptable platforms for topical peptide delivery due to their hydrated architecture and structural tunability. These systems consist of three-dimensional polymer networks capable of absorbing substantial quantities of water while maintaining cohesive integrity [41,42]. Their formation may proceed through physical crosslinking—mediated by hydrogen bonding, ionic interactions, and chain entanglement—or through covalent crosslinking, which generally yields enhanced mechanical robustness and resistance to dissolution [43,44].

The choice of polymer backbone directly determines swelling behavior, elasticity, and degradation kinetics. Natural polymers such as chitosan, alginate, collagen, and hyaluronic acid offer inherent biocompatibility and bioactivity, whereas synthetic materials including poly(vinyl alcohol) (PVA), poly(ethylene glycol) (PEG), and polyurethane (PU) provide mechanical predictability and process control [40,42]. Hybrid networks frequently combine these features, enabling modulation of porosity and crosslink density. Because water content and network tightness govern solute diffusion, these parameters critically influence AMP release profiles and matrix integrity under physiological conditions [41,43]. Thus, hydrogel performance is fundamentally linked to structural architecture rather than polymer identity alone.

#### 3.1.2. Creams and Topical Emulsions

Semisolid emulsions remain a mainstay of dermatological therapy owing to their ease of application and patient acceptability. Structurally, these systems are biphasic dispersions composed of oil and aqueous phases stabilized by surfactants and rheology modifiers [39]. Oil-in-water (O/W) or water-in-oil (W/O) configurations are selected based on desired occlusivity, hydration, and drug partitioning behavior. The physicochemical stability of emulsions depends on interfacial tension control, droplet size distribution, and viscosity regulation. Components such as fatty alcohols, plant-derived oils, glycerol, carbomers, modified celluloses, and lecithins contribute not only to texture but also to diffusion dynamics and skin interaction [45,46]. Manufacturing typically involves controlled thermal processing and shear mixing to achieve homogeneity, followed by stabilization steps including pH adjustment and preservative incorporation [45,46,47]. While often perceived as simple carriers, emulsion microstructure strongly influences AMP distribution between phases, which in turn affects stability and bioavailability at the cutaneous interface.

#### 3.1.3. Polymeric Films

Polymeric films provide a distinct structural strategy, functioning simultaneously as protective barriers and controlled-release membranes. These thin, flexible constructs adhere to the wound surface while permitting gas exchange, thereby maintaining a microenvironment conducive to healing [48]. Solvent casting remains the most frequently employed fabrication technique, whereby polymer solutions are molded and dried under regulated conditions to produce continuous layers [49,50]. Variations in polymer concentration, solvent volatility, and drying kinetics significantly affect thickness, uniformity, and mechanical resilience [51].

Both natural and synthetic polymers—including PVA, chitosan, gelatin, poly(lactic acid) (PLA), and PU—are utilized either individually or in blends to balance tensile strength and flexibility [50,52]. Plasticizers such as glycerol and PEG are commonly incorporated to reduce brittleness and enhance conformability [48]. Performance metrics—including thickness, water vapor transmission rate (WVTR), tensile resistance, and adhesion—must be calibrated to clinical requirements, particularly in wound management contexts where moisture balance and barrier protection are critical [48,52]. Film architecture therefore directly shapes the diffusion of incorporated AMPs and their local concentration gradients.

A representative example of AMP-functionalized polymeric films was reported for ultrathin chitosan films covalently conjugated with the amphibian-derived peptide Ctx(Ile^21^)-Ha through an *N*-acetylcysteine linker [53]. In this system, the peptide remains partially immobilized at the film surface rather than being freely released, allowing the material to function as a contact-active antimicrobial interface. Such spatial presentation favors electrostatic interactions between the cationic peptide domains and negatively charged bacterial membranes, promoting localized membrane disruption at the film–cell interface while minimizing peptide dilution. The conjugated films exhibited clear antibacterial activity against both Gram-positive and Gram-negative pathogens, including *S. aureus*, *Pseudomonas aeruginosa*, *Salmonella* Typhimurium, and *Escherichia coli*, whereas the unconjugated chitosan matrix showed markedly lower activity. This example illustrates how polymeric films can enhance AMP efficacy by concentrating antimicrobial activity at the surface and sustaining peptide–bacteria interactions in topical environments.

#### 3.1.4. Impregnated Wound Dressings

Impregnated dressings extend the structural complexity of topical systems by combining porous scaffolds with post-fabrication loading of active agents. These constructs frequently rely on foam matrices, semipermeable membranes, or fibrous networks that provide mechanical stability and exudate management [52]. Electrospinning is widely applied to generate nanofibrous structures, using high-voltage fields to elongate polymer solutions into continuous fibers with high surface area-to-volume ratios [54]. Materials such as PVA, cellulose acetate, and chitosan are commonly selected for their processability and compatibility, often combined with natural components to enhance absorption capacity and biocompatibility [54,55]. The resulting architecture enables selective permeability, moisture retention, and structural protection—properties closely linked to infection control and tissue regeneration [56]. In these systems, AMP loading efficiency and release behavior depend not only on polymer composition but also on fiber diameter, porosity, and surface chemistry.

Despite differences in composition and processing, these platforms converge on a shared engineering challenge: reconciling mechanical stability with biological performance. Polymer selection, solvent systems, crosslinking strategies, and manufacturing conditions collectively determine permeability, adhesion, degradation, and drug-release kinetics [57,58]. Increasingly, formulation strategies favor hybrid constructs that integrate natural biopolymers—such as chitosan, alginate, and collagen—with synthetic polymers that confer mechanical control and scalability, often enhancing biodegradability and reducing toxicity profiles [59]. A detailed understanding of matrix formation therefore provides the conceptual framework necessary for rational AMP incorporation. Structural parameters—including network density, porosity, interfacial distribution, and hydration behavior—govern peptide stability, diffusion, and therapeutic persistence. The following section examines incorporation strategies and reported biological outcomes when AMPs are integrated into hydrogels, emulsions, polymeric films, and advanced wound dressings, emphasizing how matrix architecture modulates antimicrobial performance.

### 3.2. AMPs Associated with Traditional Topical Systems

The increasing prevalence of multidrug-resistant pathogens has stimulated renewed interest in localized antimicrobial strategies, particularly in settings where high drug concentrations can be achieved without systemic exposure. In this framework, AMPs have attracted attention not merely as standalone agents but as functional components of topical delivery systems. Their relatively short sequences, tunable physicochemical properties, and, in some cases, reduced propensity for resistance selection under defined conditions have positioned them as candidates for integration into established dermatological formulations [60].

Beyond intrinsic antimicrobial activity, several AMPs display self-assembly behavior or amphipathic organization that facilitates their incorporation into structured matrices. Rather than relying on the direct application of free peptides—which may be compromised by proteolytic degradation in wound exudates—formulation within topical platforms such as creams, hydrogels, polymeric films, and impregnated dressings provides structural protection and modulates local retention [39,60]. From a translational perspective, dermal delivery remains more feasible than systemic administration, particularly for infections of the skin and soft tissue, including those predominantly caused by Gram-positive bacteria [39]. Nevertheless, topical administration does not eliminate formulation challenges. Protease-rich wound environments can rapidly inactivate free peptides, and uncontrolled diffusion may reduce effective local concentrations. Incorporation into polymeric matrices therefore serves two primary functions: enhancing peptide stability and regulating release kinetics. The resulting pharmacodynamic profile depends on matrix composition, peptide–polymer interactions, and environmental conditions at the site of application.

#### 3.2.1. Hydrogels as AMP Carriers

Hydrogels remain among the most extensively explored carriers for AMP delivery due to their hydrated microenvironment and compatibility with wound physiology [61]. Systems derived from natural polymers—such as collagen-based matrices—may additionally contribute to anti-inflammatory signaling and tissue remodeling, thereby complementing antimicrobial effects [62]. Peptide incorporation can occur through non-covalent entrapment, often driven by electrostatic interactions between cationic AMPs and negatively charged polymer backbones, or via covalent conjugation to the network to prolong retention and reduce burst release [56,63,64].

The high water content of hydrogels can preserve peptide conformation and mitigate denaturation, yet release behavior is highly dependent on network architecture. In diffusion-dominated systems, mesh size—determined by crosslinking density, polymer chemistry, and environmental stimuli—governs the mobility of incorporated peptides [39]. Swelling-responsive matrices introduce an additional variable, as hydration-induced expansion can accelerate peptide liberation. In contrast, degradation-controlled systems rely on enzymatic or hydrolytic cleavage of the polymer backbone, with release rates modulated by polymer composition, molecular weight, and structural organization [65]. Thus, AMP-loaded hydrogels should not be viewed as passive reservoirs but as dynamic systems in which matrix architecture dictates stability, bioavailability, and therapeutic persistence. Optimizing these parameters requires balancing mechanical integrity with controlled diffusion to maintain effective antimicrobial concentrations while minimizing premature depletion.

#### 3.2.2. Other Topical Platforms

Beyond hydrogel-based matrices, antimicrobial peptides have also been incorporated into conventional semisolid formulations such as creams and ointments. These vehicles remain widely used in dermatological therapy due to their manufacturing simplicity, regulatory familiarity, and capacity to provide localized drug delivery within superficial infections. From a pharmaceutical perspective, semisolid systems offer practical advantages, including ease of administration, direct contact with infected tissues, and the ability to incorporate amphiphilic or moderately lipophilic compounds within structured lipid–water dispersions. However, the performance of AMP-loaded creams and ointments is strongly influenced by physicochemical interactions between peptide molecules and the formulation matrix.

Unlike polymeric hydrogels, semisolid emulsions often present heterogeneous microenvironments composed of aqueous and lipid domains. Cationic AMPs may interact electrostatically with anionic excipients or partition preferentially within aqueous phases, which can affect both diffusion dynamics and effective bioavailability at the infection site. These interactions may either stabilize peptide conformation or, conversely, restrict diffusion toward the bacterial target. In addition, the relatively limited residence time of conventional creams and ointments on exudative or mechanically stressed skin surfaces may reduce sustained peptide exposure unless formulation strategies are implemented to enhance adhesion or viscosity. As a result, formulation optimization must consider not only peptide stability but also rheological properties, partition behavior, and local retention.

AMC-109 provides a representative example of AMP integration into traditional topical matrices. This synthetic peptide has demonstrated strong antifungal and antibacterial activity in models of onychomycosis and candidiasis, outperforming established antifungal agents such as terbinafine and amorolfine under certain experimental conditions [66,67]. Importantly, incorporation into topical vehicles did not compromise antimicrobial activity. In vitro studies including inhibition zone assays, confocal microscopy, and colony-forming unit (CFU) quantification demonstrated that AMC-109 retained potent activity against Staphylococcus aureus, including methicillin-resistant strains, when delivered through semisolid formulations [66,67,68].

Preclinical in vivo models further support the feasibility of these systems. In murine superficial infection models, topical treatment with AMC-109 formulations produced substantial reductions in bacterial burden, with some studies reporting approximately 99% decreases in S. aureus counts following treatment. The antimicrobial effect remained dose dependent and in certain cases exceeded the efficacy of commercially available topical antimicrobials used as comparators, including in MRSA-associated infections [69]. These findings suggest that semisolid vehicles can preserve peptide bioactivity while enabling localized delivery within infected tissues.

Beyond direct antimicrobial effects, several AMPs incorporated into topical matrices exhibit additional biological functions that may influence wound healing. LL-37, the only human cathelicidin identified to date, represents one of the most extensively studied peptides in cutaneous biology. In addition to its bactericidal properties, LL-37 modulates host innate immunity through interactions with pattern-recognition receptors, including Toll-like receptors, thereby influencing cytokine production, chemotaxis, and epithelial regeneration [39,70]. Such pleiotropic activity makes LL-37 particularly attractive for topical formulations designed to simultaneously control infection and promote tissue repair.

Formulation studies have explored strategies to maintain peptide stability while exploiting these regenerative properties. For example, incorporation of LL-37 into chitosan-based matrices has been investigated as a means to combine the intrinsic antimicrobial activity of the peptide with the bioadhesive and biodegradable characteristics of chitosan. In one such system, LL-37 was physically entrapped within a 2.5% (*w*/*v*) chitosan hydrogel to generate an LL-37/CS formulation with controlled degradation behavior and favorable biocompatibility [71].

In vitro evaluation demonstrated that LL-37-loaded matrices significantly inhibited *S. aureus* growth compared with chitosan alone, as assessed by CFU quantification following incubation in phosphate-buffered saline [48]. The biological performance of this formulation was further assessed in a murine deep-skin lesion model generated through prolonged magnetic compression. In this model, animals receiving LL-37 delivered through the hydrogel matrix exhibited accelerated wound closure, increased epithelial thickness, and enhanced vascularization relative to untreated controls. Histological analyses also revealed upregulation of regenerative cytokines accompanied by reduced inflammatory markers, indicating that peptide-loaded matrices may actively shape the local immune environment during healing [48].

#### 3.2.3. AMP-Loaded Dressings and Electrospun Nanofibers

In addition to hydrogel-based systems, nanofibrous and impregnated dressings have gained relevance as platforms capable of sustaining AMP release within infected wounds. An effective dressing must simultaneously maintain moisture balance, manage exudate, limit cytotoxicity, and suppress microbial proliferation [72]. Electrospinning has emerged as a versatile fabrication method in this context, enabling the generation of submicrometer fibers with tunable porosity and high surface area-to-volume ratios, properties that influence both exudate absorption and drug-release kinetics [73].

Incorporation of NP10 into a chitosan/poly(ethylene oxide) (CS/PEO) system via electrospinning produced nanofibrous membranes characterized by structural stability and controlled release behavior. In vitro evaluation of membranes containing 0.5% NP10 demonstrated sustained antibacterial activity against *S. aureus* and *E. coli*, with inhibition zones and CFU reductions persisting for up to 15 days, consistent with prolonged peptide diffusion from the fibrous network. In vivo, application to *S. aureus*-infected murine wounds accelerated tissue repair, enhanced vascularized tissue formation, and reduced bacterial burden compared with control dressings [65].

A related electrospun platform combined PVA and cellulose acetate, selected for complementary properties including water solubility, processability, and chemical stability [74]. In this system, two peptides were incorporated through distinct strategies: pexiganan was covalently immobilized using a PEG2–maleimide spacer to ensure stable surface presentation, whereas Tiger-17 was physically adsorbed to preserve conformational flexibility and bioactivity. This dual-incorporation design sought to integrate antimicrobial and hemostatic functions within a single dressing. Antibacterial testing against *S. aureus* and *Pseudomonas aeruginosa* confirmed significant growth inhibition, primarily attributable to pexiganan, while Tiger-17 shortened coagulation time and maintained cytocompatibility in L929 fibroblasts. The differentiation of covalent versus non-covalent loading strategies highlights how peptide–matrix interactions influence release behavior and functional performance. Rather than functioning merely as reservoirs, electrospun dressings actively shape AMP pharmacodynamics through fiber diameter, porosity, surface chemistry, and degradation rate. Control over these parameters is essential to avoid burst release or premature peptide depletion, challenges that remain relevant for clinical translation.

#### 3.2.4. Polymeric Films Incorporating AMPs

Polymeric films constitute a structurally distinct topical format, combining barrier function with localized drug delivery. Clinical applicability depends on balancing flexibility, adhesion, ease of removal, and exudate management while maintaining semi-occlusive properties conducive to healing [75]. Incorporation of AMPs into film matrices can enhance stability by shielding peptides from enzymatic degradation and enabling controlled diffusion across the wound interface [53]. Films containing ε-poly-L-lysine (ε-PLL), a peptide produced by *Streptomyces albulus*, exemplify this approach. ε-PLL exerts antibacterial activity primarily through electrostatic interaction with negatively charged bacterial membranes. When integrated into chitosan/PVA films, FTIR analysis supported homogeneous peptide distribution, and mechanical testing indicated adequate tensile strength and flexibility compatible with wound coverage [69].

Biological evaluation demonstrated increased antibacterial activity against *S. aureus* and *E. coli* compared with polymer-only controls, as assessed by agar diffusion assays. The ε-PLL-containing films also displayed balanced porosity and minimal cytotoxicity toward 3T6-Swiss fibroblasts, supporting biocompatibility [69]. These findings indicate that film-based systems can preserve AMP activity while providing structural protection and localized delivery. Despite promising preclinical results, polymeric films and electrospun dressings share translational challenges, including reproducibility of peptide loading, stability during storage, and scale-up under Good Manufacturing Practice (GMP) conditions. While hydrogels and nanofibrous dressings often demonstrate favorable sustained-release profiles, robust clinical validation remains limited across all platforms. Thus, AMP-based topical systems should be considered evolving therapeutic constructs that integrate antimicrobial control with wound-repair modulation rather than fully established clinical solutions. The AMPs discussed in this section are summarized in the accompanying Table 1 alongside additional examples reported in the literature.

### 3.3. AMPs Encapsulated in Nanostructured Formulations

Although antimicrobial peptides demonstrate broad-spectrum activity, their therapeutic deployment remains constrained by proteolytic susceptibility, short biological half-life, and manufacturing costs that complicate large-scale production [80]. These limitations have motivated the exploration of nanostructured delivery systems as protective and pharmacokinetic-modulating platforms rather than as simple carriers. In the context of AMP delivery, nanosystems are not relevant merely because of their size, but because nanoscale architecture enables control over surface area, diffusion pathways, and peptide–matrix interactions. Such structural control can enhance resistance to enzymatic degradation, modulate release kinetics, and influence biodistribution profiles [80,81]. Nanostructures within the 1–1000 nm range have been widely investigated in drug delivery, including sustained-release depots and systems capable of traversing biological barriers. Fabrication strategies typically involve either entrapment of the therapeutic agent during particle formation or adsorption onto preformed nanocarriers, with polymer composition, crosslinking density, and surface chemistry dictating stability and release behavior [81].

For AMPs specifically, nanosystems provide three principal advantages: physical shielding against proteases, reduction in rapid systemic clearance, and capacity for controlled or stimuli-responsive release. The choice of matrix material—whether polymeric, lipid-based, or hybrid—determines peptide loading efficiency, structural integrity, and interaction with the biological microenvironment. Importantly, encapsulation differs mechanistically from surface conjugation. While conjugated systems rely on covalent or electrostatic anchoring of the peptide to the carrier exterior, encapsulated systems entrap or complex the peptide within the nanostructure core or network, thereby prioritizing protection and gradual liberation. Accordingly, this section focuses on nanosystems in which AMPs are physically encapsulated or electrostatically complexed within nanostructured matrices. These strategies aim to enhance peptide stability in protease-rich environments, prolong local retention, and maintain bioactivity during sustained release, without altering the primary sequence through covalent attachment.

#### 3.3.1. Lipid Nanocarriers

An example of an option for nanoformulation of AMPs is lipid nanocarriers (composed by solid–solid lipid mixture or by solid–liquid lipid mixture), a class of nanoformulations that encompasses small spherical vesicles with high surface area, composed of ionizable lipids, such as liposomes, micelles, liquid crystalline nanoparticles and nanostructured lipid carriers [82]. Liposomes are among the most extensively studied nanosystems, consisting of spherical nanometric vesicles with one or multiple concentric bilayers of natural or synthetic amphiphilic lipid molecules, primarily cholesterol and phospholipids, enclosing an aqueous core; the amphiphilic nature of these lipids enables self-assembly with hydrophilic head groups oriented toward the aqueous phases and hydrophobic tails aligned within the bilayer, and due to their biocompatibility and biodegradability, liposomal drug delivery systems are designed to modulate the pharmacokinetic and pharmacodynamic properties of their cargo to overcome limitations such as low efficacy, poor bioavailability, and high toxicity, which is the case of several antimicrobial peptides of scientific interest [80].

Omiganan is a synthetic cationic peptide from the cathelicidin family, composed of 12 amino acid residues, and is recognized for its broad antimicrobial activity, including against *S. aureus*, making it a promising candidate for wound healing applications. Its mechanism of action involves depolarization of the cytoplasmic membrane, leading to cellular disruption and death, as well as a dose-dependent inhibition of protein, DNA, and RNA synthesis in *S. aureus*. In a study by Javia et al., Omiganan was incorporated into a liposomal gel and compared with conventional formulations (gel and lotion) in mouse models of atopic dermatitis and psoriasis; the liposomal system provided controlled peptide release, improved skin permeation, and exhibited anti-inflammatory activity by reducing pro-inflammatory cytokine levels in the lesions [83]. Accordingly, the relevance of liposomes in AMP delivery for wound healing is supported by a substantial body of studies, some of them presented in this work.

In a study conducted by Changsan et al., the antimicrobial peptide P1-AMP (Table 2), isolated from *Brevibacillus* sp., demonstrated significant activity against methicillin-resistant *S. aureus* strains and standard *S. aureus* strains, and its encapsulation in a liposome-loaded hydrogel, composed of L-a-phosphatidylcholine and cholesterol in a ratio of 18:1, signicantly enhanced the permeability of P1-AMP through the skin compared to P1-AMP without encapsulation in the liposome, in addition to the increase in the peptide stability when stored at 30 °C and 40 °C with high humidity for at least 6 months under the studied conditions [84].

Still on the subject of liposomes, in a study conducted by Gelen-Gungor et al., the antimicrobial activity of the AMP nisin, a generally recognized as safe peptide which has a particular effectiveness in the inhibition of *S. aureus* (the most common pathogen of dermal infections), was explored in combination with azithromycin (a semi-synthetic macrolide antibiotic derived from erythromycin), in azithromycin- and nisin-loaded liposome formulations. According to the authors, the mixing of lipids in the liposome structure with cellular lipids in the stratum corneum enhanced skin moisturization and the penetration of nisin, and the combined liposomal formulation exhibited superior antimicrobial/antibiofilm activities and biocompatibilities when compared to azitromicin-loaded liposomes, demonstrating how this type of drug delivery nanosystem can enhance the activity of antimicrobial compounds [85]. Table 2 presents more examples of studies using liposomes.

Another category of lipid nanocarriers is solid lipid nanoparticles (SLNs), investigated by Fumakia & Ho, in a study that evaluated the synergistic activity of the LL37 peptide, an endogenous host-defense antimicrobial peptide, with serpin A1, an elastase inhibitor with wound healing properties, in a topical combination nanomedicine designed for controlled and sustained delivery. The nanoparticles were synthetized by a water/oil/water double emulsion technique, which offered protection to the peptide and A1. The tests with this formulation resulted in acceleration of the wound healing process by promoting wound closure in BJ fibroblast cells and keratinocytes, which can synergistically enhance antibacterial activity and confer anti-inflammatory activity by the reduction in lipopolysaccharides (LPS) and upregulated inflammatory cytokines in the cells mentioned [89].

Another study addressing the activity of the AMP LL37 in nanosystems was reported by García-Orue et al., in which LL37 was encapsulated in nanostructured lipid carriers (NLCs), a delivery system composed of a mixture of solid and liquid lipids that enhances the solubility of lipophilic compounds [82,87]. The NLCs, produced using the melt–emulsification method, effectively protected the peptide without compromising its activity, as demonstrated by immunomodulatory and antimicrobial in vitro assays. Furthermore, an in vivo full-thickness wound healing assay in mice showed that LL37-loaded NLCs significantly accelerated the healing process, improving wound closure, re-epithelialization, and resolution of the inflammatory phase when compared to the same concentration of free LL37 in solution [87].

#### 3.3.2. Polymeric Nanoparticles

Polymer-derived nanomaterials are widely employed in AMP nanoformulations, as they consist of small particles that enhance AMP solubility and protect them from protease degradation, while also enabling controlled and sustained release and site-specific accumulation [82,93]. AMPs can be adsorbed, dissolved, encapsulated, or covalently attached to polymers, with release occurring primarily through diffusion, and these systems additionally display a broad spectrum of antibacterial activity with a low tendency for inducing bacterial resistance [94,95]. Usually, polymeric nanoparticles (PNs) are constituted by natural polysaccharides, such as chitosan, pectin and alginate, or synthetic biopolymers such as polyesters, like poly-lactic acid (PLA) and poly-lactide-co-glycolide (PLGA). Multiple reports have shown that chitosan (CS) is the polymer most often applied in delivery systems for AMPs, especially in cases of wound injuries, due to their intrinsic antimicrobial activity and their mucoadhesive properties [63,94].

Focusing again on LL37, in a study by Yang and collaborators, the AMPs were encapsulated with CS hydrogel and tested in a mouse model for wound injuries. The resulting LL37 CS hydrogel significantly reduced the area of pressure ulcers and enhanced epithelial thickness as well as the density of newly formed capillaries when compared with free LL37 [63]. Still addressing CS polymeric nanosystems carrying LL37, in a study conducted by Rashki et al., LL37-loaded chitosan nanoparticles were tested against methicillin-resistant *S. aureus* (MRSA) and showed to prolong the antibacterial activity of the AMP by increasing its half-life, in addition to its 68% biofilm formation inhibition, compared to the analyses of LL37 alone [90].

As an example of nanosystems using synthetic biopolymers, a study by Üstün & Örtücü can be mentioned, in which the aforementioned AMP nisin were loaded to PLGA nanoparticles and tested against planktonic *S. aureus* and *S. aureus* biofilms. The results revealed that the nanoformulation was more efficient in eradicating the bacteria and in inhibiting biofilm formation compared to the use of free nisin, showing it to be a promising strategy to be applied in the treatment of wound infections [86].

#### 3.3.3. Dendrimers

Dendrimers are hyperbranched polymeric nanostructures (2–5 nm) composed of a core, branching layers, and terminal functional groups, where higher generations provide more binding sites that can be functionalized with targeting moieties or multiple drug molecules to enhance site-specific delivery and interactions with bacterial cells. Their globular architecture also creates nanodomains that enable drug encapsulation [81]. A study carried out by Zhang et al. described the incorporation of a human kininogen-derived peptide, DPK-060, into dendritic nanogels embedded into poloxamer gel, creating a nanosystem that improved the AMP antimicrobial efficiency in inhibiting the growth of *S. aureus*, observed in in vitro and in vivo anti-infection tests on ex vivo pig skin and in vivo mouse infection models, in addition to the slower release when compared to the gel formulation with free DPK-060 [91].

#### 3.3.4. Other Types of Nanomaterials

Cubosomes are a type of liquid crystalline nanoparticle that consist of folded lipid bilayers arranged in a three-dimensional structure with interconnected aqueous channels. Their complex amphipathic architecture allows the incorporation of hydrophilic drugs within the aqueous channels, hydrophobic drugs in the lipid bilayers, and amphiphilic drugs at the bilayer–water interface [96]. Another type of nanomaterial that can be mentioned is nanogels, which is approached in Obuobi et al.’s work, that described the sustained release of the AMP L12 encapsulated in DNA nanogel in *S. aureus* models of infectious bacterial keratitis, which resulted in the reduction in clinical symptoms and microbial burden of the bacteria, attested by the progressive decrease in corneal thickness and quantitative analysis of bacterial viability [92].

Cyclodextrins have been widely explored in recent years for AMP delivery. These molecules are cyclic oligosaccharides formed by multiple dextrose units connected through α-1,4-glucosidic linkages, creating a hollow cavity that is hydrophobic inside and hydrophilic outside, which confers solubility, biocompatibility, and structural stability [80]. In a study by Monfared et al., a nanosystem based on cyclodextrin nanosponges synthesized with carbonyldiimidazole (CDI) and pyromellitic dianhydride (PMDA) was used to deliver the nisin Z peptide. This system enhanced the antimicrobial activity of the AMP against *S. aureus* and *E. coli*, while also preventing nisin degradation by pepsin in cooked chicken meat, highlighting its potential as a promising strategy for the treatment of wound infections commonly caused by these pathogens [97].

Beyond organic and lipid-based systems, inorganic nanocarriers provide an alternative strategy for improving intracellular delivery of antimicrobial peptides. In this context, mesoporous silica nanoparticles (MSNs) have emerged as versatile platforms capable of enhancing peptide stability, cellular uptake, and controlled release. Using a machine learning-guided design strategy, the peptide DC05 was identified and subsequently loaded into MSNs, followed by surface functionalization with tuftsin, a macrophage-targeting cell-penetrating peptide. This system (MSN-CPP@DC05) improved antimycobacterial activity against *Mycobacterium tuberculosis*, reducing MIC values relative to the free peptide while maintaining low cytotoxicity and minimal hemolysis [98]. Importantly, functionalization enhanced intracellular delivery into macrophages, a key barrier in tuberculosis therapy. Mechanistic analyses combining scanning electron microscopy and molecular dynamics supported a membrane-disruptive mode of action, while in vivo *Galleria mellonella* assays confirmed a favorable safety profile. These findings highlight that inorganic nanocarriers, when combined with rational peptide design and targeting ligands, represent a complementary strategy to lipid-based systems for addressing intracellular infections.

### 3.4. Intra- and Extramolecular Conjugation Strategies of Antimicrobial Peptides

AMPs display rapid bactericidal activity and broad-spectrum efficacy; however, their clinical translation is frequently constrained by limited proteolytic stability, cytotoxicity, and unfavorable pharmacokinetic behavior. Structural modification has therefore become a central strategy to optimize peptide performance while preserving antimicrobial function. These modifications can be broadly divided into intramolecular approaches, which alter the peptide backbone or side chains, and extramolecular approaches, which introduce additional functional moieties to expand peptide behavior.

#### 3.4.1. Intramolecular Conjugation of Antimicrobial Peptides

Intramolecular modification of antimicrobial peptides involves the introduction of chemical or post-translational changes within the primary sequence to modulate electrostatics, conformational dynamics, membrane interaction, and proteolytic susceptibility. Rather than acting as simple decorative alterations, these modifications frequently redefine peptide–membrane energetics and structural stability. Phosphorylation represents one of the most biologically established intramolecular modifications. The addition of phosphate groups to serine, threonine, or tyrosine residues (O-phosphorylation) introduces negative charge and can alter peptide folding and target interaction [99]. O-linked phosphorylation is chemically stable and increasingly explored in peptide engineering. In contrast, *N*-phosphorylation at histidine, lysine, or arginine is intrinsically labile, which complicates synthetic application despite growing recognition of its regulatory importance [100,101]. Functional dependence on phosphorylation is exemplified by salivaricin 10, a ribosomally synthesized and post-translationally modified peptide from Streptococcus salivarius, in which antimicrobial, antibiofilm, and immunomodulatory activities require the phosphorylated state [102].

Glycosylation introduces a distinct layer of modulation through covalent attachment of carbohydrate moieties. Depending on linkage chemistry and glycan composition, glycosylation can influence aqueous solubility, steric shielding, membrane affinity, and resistance to proteolysis [103,104,105]. O-glycosylation at serine or threonine residues is widely characterized in mammalian systems, where it regulates diverse physiological processes including oncogenic signaling [106,107]. In bacteriocins such as enterocin F4-9 from Enterococcus faecalis, removal of the glycan moiety markedly reduces antimicrobial potency, underscoring its structural relevance [108,109].

*N*-glycosylation at asparagine within the canonical Asn-X-Ser/Thr motif has been leveraged to generate glycosylated analogs of tyrocidine A active against Bacillus subtilis, methicillin-resistant *S. aureus*, and vancomycin-resistant *Enterococcus* species [14,104]. Alternative linkages expand structural diversity: S-glycosylation at cysteine residues enhances resistance to hydrolytic cleavage, as demonstrated in glycocin F analogs with bacteriostatic activity exceeding that of both native and O-glycosylated forms [110]. C-glycosylation at tryptophan residues has yielded glycotriazole peptides with improved antifungal performance relative to the parent scaffold [111]. Nevertheless, glycosylation does not universally enhance activity; steric hindrance or altered membrane interaction can attenuate potency, emphasizing the necessity of sequence- and target-specific optimization [107].

Acetylation and methylation further illustrate how subtle chemical changes recalibrate AMP behavior. *N*-terminal acetylation—common among antimicrobial peptides and largely irreversible—can modify insertion depth and membrane perturbation capacity [112,113]. For example, *N*-terminal acetylation of L1A enhanced membrane destabilization, likely by promoting deeper bilayer penetration [114]. *N*-methylation influences backbone rigidity, metabolic stability, and lipophilicity [115]. In gramicidin S, selective *N*-methylation disrupted internal hydrogen bonding and reduced amphiphilicity, attenuating hemolysis while preserving antibacterial activity, particularly when modifications targeted β-sheet and β-turn regions [116]. These observations illustrate how intramolecular modifications can decouple antimicrobial potency from host toxicity.

Among structural strategies, cyclization is especially effective in enhancing conformational constraint and protease resistance. Head-to-tail cyclization or side chain-mediated linkages reduce conformational entropy and protect against enzymatic cleavage [117]. Stapled analogs of magainin 2 exhibited improved antimicrobial efficacy with diminished hemolytic effects [118], while the cyclic peptide CE-05, derived from WLBU2, maintained bactericidal activity after prolonged proteolytic exposure [119]. By restricting flexibility, cyclization stabilizes bioactive conformations and prolongs functional persistence under physiological conditions.

The diversity of intramolecular modifications applied to antimicrobial peptides highlights how relatively subtle chemical alterations can produce substantial functional consequences. Changes in charge distribution, backbone rigidity, hydrophobicity, or steric shielding may influence peptide–membrane interactions, proteolytic stability, cytotoxicity, and overall antimicrobial potency. Importantly, these effects are highly context dependent and often vary according to peptide sequence, structural scaffold, and microbial target. To facilitate comparison across different chemical strategies, representative examples of phosphorylation, glycosylation, acetylation, methylation, and cyclization applied to antimicrobial peptides are summarized in Table 3, together with their reported antimicrobial activities, design objectives, and observed biological outcomes.

#### 3.4.2. Extramolecular Conjugation of AMPs

Extramolecular conjugation strategies introduce external functional units to modulate peptide distribution, stability, and selectivity. PEGylation improves solubility, circulation time, and toxicity profiles but may compromise antibacterial activity when long polymer chains are employed, favoring the use of short PEG moieties [125,126]. Lipidation, achieved through attachment of fatty acid chains to *N*-terminal residues or lysine side chains, enhances membrane affinity and antimicrobial potency, although excessive hydrophobicity is closely associated with increased cytotoxicity [125,127].

Peptide–antibiotic conjugates combine membrane-active peptides with intracellularly acting antibiotics, enabling synergistic mechanisms. Conjugation of lipidic paenipeptin C′ analogs to ciprofloxacin generated constructs capable of membrane permeabilization followed by intracellular drug release, resulting in enhanced antibacterial activity relative to unconjugated peptides [128,129,130].

To overcome permeability barriers, particularly in Gram-negative bacteria, siderophore–peptide conjugates exploit bacterial iron uptake pathways to enable active transport of antimicrobial agents into the cytosol [131,132,133,134]. Antibody–peptide conjugates aim to further restrict antimicrobial activity to defined targets, improving selectivity and reducing systemic toxicity. Although still limited by cost and early developmental status, recent advances indicate potential for highly targeted antimicrobial interventions.

Collectively, these extramolecular conjugation strategies illustrate how the pharmacological behavior of antimicrobial peptides can be reshaped through the addition of external functional modules. By modifying physicochemical properties such as hydrophobicity, steric shielding, receptor recognition, or intracellular delivery pathways, these conjugates extend the functional repertoire of native AMPs beyond direct membrane disruption. Importantly, each conjugation approach presents distinct advantages and limitations depending on the intended therapeutic context, microbial target, and desired pharmacokinetic profile. To facilitate comparison across these different engineering strategies, representative examples of PEGylation, lipidation, antibiotic–peptide conjugation, siderophore-mediated delivery, and antibody–peptide targeting are summarized in Table 4, together with their antimicrobial activities, design objectives, and observed biological outcomes.

**Figure 2:**
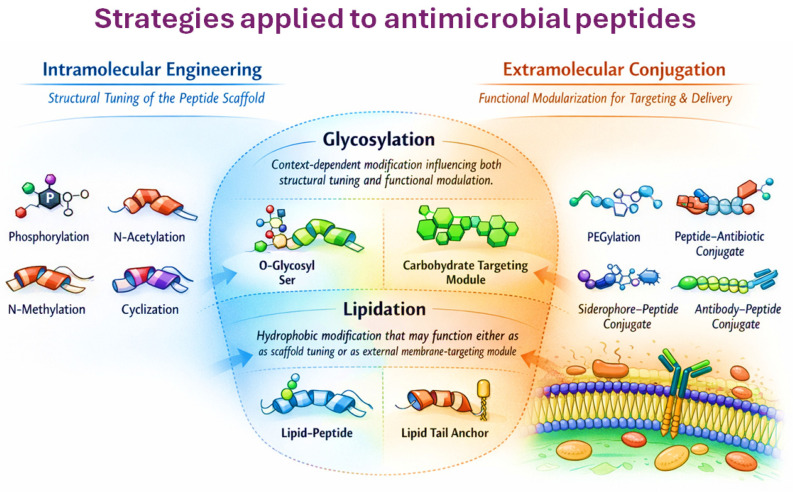
Intramolecular and extramolecular engineering strategies applied to antimicrobial peptides. (**Left panel**): Intramolecular modifications embedded within the peptide scaffold, including phosphorylation, *N*-acetylation, *N*-methylation, and cyclization, which primarily tune peptide physicochemical properties such as charge distribution, conformational stability, protease resistance, and membrane interaction. (**Right panel**): Extramolecular conjugation strategies involving the attachment of external functional modules, including PEGylation, peptide–antibiotic conjugates, siderophore–peptide conjugates, and antibody–peptide conjugates, which enhance pharmacokinetics, targeting, and intracellular delivery. (**Central region**): Glycosylation and lipidation represent context-dependent modifications that may function either as intramolecular physicochemical tuning or as extramolecular modules contributing to targeting or membrane anchoring. This schematic illustration was generated using Chat GPT 5/AI-assisted graphical tools and subsequently curated and scientifically validated by the authors.

Following the comparative overview presented in Table 4, Figure 2 provides a conceptual framework that distinguishes intramolecular engineering from extramolecular conjugation strategies applied to antimicrobial peptides. In the left panel, intramolecular modifications refer to chemical alterations embedded within the peptide scaffold that directly influence the intrinsic physicochemical properties of the sequence. These include phosphorylation, *N*-acetylation, *N*-methylation, and cyclization, which primarily affect charge distribution, conformational stability, proteolytic susceptibility, and peptide–membrane interactions without introducing independent functional entities [142,143]. By contrast, the right panel illustrates extramolecular conjugation approaches in which antimicrobial peptides are covalently linked to external functional modules that extend their pharmacological capabilities. Examples include PEGylation to modulate solubility and circulation time, peptide–antibiotic conjugates that combine membrane permeabilization with intracellular drug action, siderophore–peptide constructs that exploit bacterial iron uptake pathways to overcome permeability barriers in Gram-negative organisms, and antibody–peptide conjugates designed to improve pathogen-specific targeting [144]. Importantly, the central region of the figure highlights modifications that can occupy an intermediate conceptual space between these two categories. Glycosylation, illustrated as O-linked glycosylation of serine residues (O-glycosyl Ser), represents a covalent attachment of carbohydrate moieties to hydroxyl-containing residues such as serine or threonine. Depending on the design strategy, this modification may function as an intramolecular structural adjustment affecting peptide solubility and stability, or alternatively as a carbohydrate-based recognition module that contributes to targeting and molecular interactions. A similar dual interpretation applies to lipidation. Attachment of fatty acid chains can operate as a physicochemical tuning mechanism that increases hydrophobicity and membrane affinity, but in other contexts the lipid moiety behaves as an external anchoring module that promotes membrane targeting or facilitates delivery across biological barriers. By positioning glycosylation and lipidation at this conceptual interface, the figure emphasizes that peptide engineering strategies often exist along a continuum between structural tuning of the peptide scaffold and functional modularization through external conjugation [104,145].

### 3.5. In Vivo and Ex Vivo Evaluation of Topical AMP Formulations

Despite their potent antimicrobial activity, the clinical development of AMPs remains limited by several challenges, particularly related to proteolytic instability, cytotoxicity, and route of administration. When administered systemically, AMPs are prone to rapid degradation and may induce adverse effects, including nephrotoxicity and neurotoxicity, which significantly restrict their therapeutic window [9]. In this context, topical delivery has emerged as a rational alternative, enabling localized treatment at the site of infection while minimizing systemic exposure and associated toxicity, particularly in cutaneous and soft tissue infections [146].

**Figure 3:**
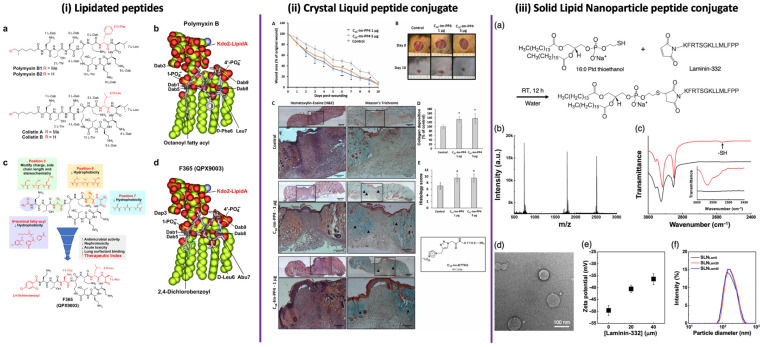
Multiscale strategies involving lipid-modified antimicrobial systems. (**i**) Structure-guided optimization of polymyxin lipopeptides. (**a**) Chemical structures of polymyxin B1/B2 and colistin A/B, highlighting residue variability. (**b**) Molecular model of polymyxin B bound to Kdo2-lipid A, illustrating key electrostatic and hydrophobic interactions. (**c**) Structure-guided modifications at the *N*-terminal fatty acyl chain and positions 3, 6, and 7 aimed at reducing hydrophobicity, nephrotoxicity, acute toxicity, and lung surfactant binding, leading to the optimized derivative F365 (QPX9003). (**d**) Molecular model of F365 bound to Kdo2-lipid A, showing preservation of critical binding interactions despite reduced hydrophobicity. Reproduced from Roberts et al., 2022 [147], *Nature Communications* (CC BY 4.0). (**ii**) In vivo evaluation of peptide–lipid supramolecular systems in wound healing. (**A**) Time-course analysis of wound closure following peptide treatment and Results are presented as means ± SD; * *p* < *0.05* ** *p* < *0.01* compared to control. (**B**) Representative macroscopic images of wound healing progression. (**C**) Histological evaluation using hematoxylin and eosin (H&E) and Masson’s trichrome staining. (**D**) Quantification of collagen deposition. (**E**) Histopathological scoring of tissue regeneration and data are expressed as mean ± SD of 4 animals (each condition of the C16-Im-PP4 treatment) and mean ± SD of 3 animals (control). The student *t*-test was used for the represented comparisons. * *p* < *0.05*. Reproduced from Gomes et al., 2025 [150], *Biochemical and Biophysical Research Communications* (Elsevier). (**iii**) Lipid–peptide conjugates for nanostructured delivery. (**a**) Chemical conjugation strategy between a lipid moiety (phospholipid derivative) and a laminin-derived peptide via thiol–maleimide chemistry. (**b**) Mass spectrometry confirming successful conjugation. (**c**) FTIR spectra indicating characteristic functional group transitions after conjugation. (**d**) Transmission electron microscopy (TEM) showing nanoparticle morphology. (**e**) Zeta potential measurements as a function of peptide density. (**f**) Particle size distribution profiles. Reproduced with permission from Kang et al., 2025 [151], *ACS Applied Nano Materials*.

A paradigmatic advance in lipid-modified antimicrobial peptides is exemplified by the synthetic lipopeptide APR46 (Figure 3i), marking a milestone in the rational optimization of polymyxin-derived scaffolds [147]. Importantly, the lipid moiety in APR46 should not be interpreted as an extramolecular conjugation, but rather as an intrinsic structural feature of the molecule, retained and refined through semisynthetic modification of the polymyxin framework. This optimization preserves membrane-targeting capability while improving the overall therapeutic profile. In contrast, panels (ii) and (iii) illustrate fundamentally different extrinsic strategies for lipid incorporation. In (ii), lipid phases act as supramolecular extracellular environments, as observed in peptide–lipid complexes, where lipid organization modulates peptide presentation, tissue interaction, and biological outcome without covalent modification. In (iii), lipid moieties are covalently introduced through site-specific chemical conjugation, enabling controlled self-assembly into nanostructured delivery systems. Together, these examples demonstrate that lipidation operates across distinct mechanistic levels, ranging from intrinsic pharmacophoric elements to supramolecular environments and fully extramolecular conjugation strategies.

Polymyxins illustrate how strategic peptide modification can convert a toxic, poorly drug-like natural lipopeptide into a clinically useful last-line antibiotic class. Starting from a non-ribosomal peptide scaffold, systematic changes to the *N*-terminal fatty acyl group and selected side chains allowed fine-tuning of charge, hydrophobicity, and conformation, thereby optimizing interactions with lipid A while progressively decoupling antibacterial potency from nephrotoxicity and acute toxicity. As highlighted, this evolution from “raw” peptide metabolite to refined polymyxin derivatives underscores the broader principle that rational, position-specific modification of peptide antibiotics can unlock new therapeutic space, rescue old scaffolds, and guide the design of safer next-generation lipopeptides [10].

To support the safe and effective development of topical AMP formulations, in vivo and ex vivo models have become essential components of preclinical evaluation. These models allow simultaneous assessment of key parameters, including skin penetration, peptide stability, release kinetics, cytotoxicity, local immune responses, antimicrobial efficacy, and wound healing under conditions that more closely resemble the clinical environment than conventional in vitro assays [148,149].

#### 3.5.1. Ex Vivo Models for Topical AMP Evaluation

Ex vivo models are based on excised tissues or organs obtained from living organisms and maintained under controlled laboratory conditions that preserve the structural integrity and physiological relevance [96,149,152]. In the context of topical formulations, ex vivo skin models provide a realistic barrier that enables the evaluation of stratum corneum penetration, peptide retention, controlled release, and local toxicity, parameters that cannot be adequately captured using simplified in vitro systems [149]. Beyond barrier function, ex vivo approaches have also been used to investigate regenerative and immunomodulatory effects. Costa and co-workers demonstrated that the AMPs clavanin A and mastoparan-MO promoted keratinocyte migration and increased expression of wound healing markers in ex vivo skin models, suggesting a direct contribution to tissue regeneration in addition to antimicrobial activity [153]. Similarly, Ross et al. developed peptide-based hydrogels and assessed their skin adhesion strength and wound-closure capacity using ex vivo models, parameters of direct relevance for the clinical performance of topical dressings [154].

Although still less frequently employed than in vivo models, ex vivo systems occupy an increasingly important role in preclinical research. By preserving the architectural and functional complexity of skin tissue, these models offer improved biological relevance compared with in vitro assays, while substantially reducing ethical concerns associated with animal experimentation [152,155]. A representative example is provided by the work of Boge et al., who evaluated the antimicrobial activity of LL-37 encapsulated in cubosomes using porcine skin infected with *S. aureus*. The ex vivo model demonstrated that encapsulated LL-37 retained bactericidal activity, exhibited resistance to pathogen-derived proteases, and did not induce cytotoxic effects in keratinocytes. Importantly, these findings supported and refined previous in vitro observations, reinforcing the value of ex vivo models as predictive tools for subsequent in vivo evaluation of topical AMP formulations [96].

#### 3.5.2. In Vivo Models for Efficacy and Wound Healing Assessment

In vivo studies, conducted in living organisms such as rodent models, remain indispensable for evaluating AMP formulations under fully integrated physiological conditions. These models enable simultaneous analysis of antimicrobial efficacy, immune response, tissue regeneration, and safety, thereby providing critical data for progression toward clinical studies [17,156]. Nevertheless, in vivo experimentation requires strict adherence to ethical guidelines and animal welfare regulations to ensure responsible and reproducible research [60]. Jung Kim et al. evaluated the synthetic AMP SHAP1, designed to overcome common limitations of natural AMPs such as susceptibility to proteolytic degradation. In a murine excisional wound model infected with *S. aureus*, topical application of SHAP1 was directly compared with the endogenous human peptide LL-37. While both peptides reduced bacterial burden, SHAP1 demonstrated superior wound healing performance, as evidenced by faster wound closure and more uniform tissue repair, highlighting its therapeutic potential as a topical antimicrobial with regenerative properties [148].

The AMP RRP9W4N represents another example of successful in vivo evaluation. Atefyekta et al. incorporated this peptide into a mesoporous hydrogel composed of quaternized chitosan and methacrylated gelatin for the treatment of infected skin wounds. In a murine model of *S. aureus*-infected dorsal wounds, animals treated with the AMP-loaded hydrogel exhibited significantly reduced bacterial counts, accelerated wound closure, and enhanced collagen deposition compared with untreated and hydrogel-only controls. Histological analyses further confirmed improved tissue regeneration and reduced inflammatory burden, demonstrating the synergistic effect of peptide activity and hydrogel delivery [78]. In vivo wound healing activity has also been reported for endogenous human β-defensins. Takahashi et al. investigated the topical application of hBD-3 in a murine wound model and observed increased recruitment of neutrophils, macrophages, and fibroblasts, along with enhanced angiogenesis and elevated expression of growth factors such as FGF, PDGF, and VEGF. These findings underscore the dual antimicrobial and immunomodulatory roles of AMPs in tissue repair [157].

Håkansson et al. assessed the AMP DPK-060 formulated in a poloxamer gel using both in vivo and ex vivo models. In mice infected with *S. aureus*, topical treatment with DPK-060 significantly reduced bacterial survival compared with the placebo, showing a clear dose–response relationship and efficacy comparable to conventional topical antibiotics. Complementary ex vivo experiments using porcine skin confirmed sustained antimicrobial activity and reinforced the translational relevance of this formulation [149]. More complex delivery systems have also been evaluated in vivo. Yu et al. investigated electrospun chitosan/polyethylene oxide nanofibrous dressings containing the AMP NP10 in a rat model of *S. aureus*-colonized wounds. Treatment resulted in significant wound-size reduction over a 14-day period, confirming not only antimicrobial efficacy and biocompatibility but also the capacity of AMP-loaded dressings to actively promote wound healing [65].

Beyond bacterial infections, topical AMP formulations have been explored for fungal skin diseases. Zou et al. evaluated the cationic α-helical peptide ACP5 in a guinea pig model of dermatophytosis caused by Trichophyton mentagrophytes. ACP5 demonstrated potent fungicidal activity and, notably, a low propensity for resistance development compared with conventional antifungal agents, highlighting its potential for chronic or recurrent topical applications [158]. Collectively, these studies demonstrate that the development of topical AMP formulations is intrinsically linked to robust preclinical evaluation using complementary in vivo and ex vivo models. While in vivo systems remain the cornerstone for assessing integrated antimicrobial, immunological, and regenerative responses, ex vivo models provide valuable mechanistic and translational insight while reducing animal use and enabling controlled experimental conditions. Despite their clear advantages, ex vivo approaches remain underrepresented in the literature, particularly for topical AMP formulations, representing a substantial opportunity for methodological advancement. Strategic integration of in vitro, ex vivo, and in vivo models—combined with rational formulation design aimed at enhancing stability, controlled release, and local safety—will be essential to accelerate the clinical translation of AMPs as effective therapies for resistant cutaneous infections.

The experimental studies discussed above illustrate the range of animal models currently used to evaluate the antimicrobial and wound healing potential of AMP-based formulations under physiologically relevant conditions. Differences in host species, infection models, and therapeutic strategies often influence both antimicrobial outcomes and tissue regeneration dynamics. For clarity, representative in vivo studies investigating antimicrobial peptides across different animal models and infection settings are summarized in Table 5.

## 4. Perspectives and Future Directions

The accumulated evidence suggests that the therapeutic performance of antimicrobial peptides cannot be interpreted independently of formulation architecture and biological context. Proteolytic instability, concentration-dependent cytotoxicity, and inconsistent in vivo efficacy frequently arise not from intrinsic limitations of peptide scaffolds but from mismatches between peptide physicochemical properties and their delivery environment. Several peptides that exhibit limited stability or moderate activity in aqueous systems display markedly improved antimicrobial performance and tolerability when integrated into structured topical matrices such as hydrogels, nanofibrous dressings, or polymeric carriers. This observation reflects an important conceptual shift in the field: the clinical success of AMPs may depend less on the continuous discovery of new sequences and more on the rational engineering of peptide–formulation systems capable of stabilizing bioactive conformations and sustaining local activity within complex biological environments.

Chemical modification strategies further support this transition toward functional optimization. Intramolecular modifications—including cyclization, methylation, and glycosylation—and extramolecular conjugation approaches involving polymers, lipids, antibiotics, or targeting ligands have significantly expanded the functional repertoire of AMPs. These strategies can increase proteolytic resistance, modulate membrane affinity, reduce host toxicity, and improve pharmacokinetic behavior. However, the evaluation of such modifications often remains restricted to planktonic MIC measurements, which provide only a limited representation of therapeutic potential. In topical infections in particular, antimicrobial performance is strongly influenced by biofilm architecture, host-derived proteases, inflammatory mediators, and the physicochemical properties of the wound microenvironment. Consequently, future evaluation frameworks must integrate parameters such as antibiofilm activity, peptide persistence in protease-rich environments, immunomodulatory signaling, and compatibility with tissue repair processes.

Another major challenge in AMP research lies in the translational gap between simplified laboratory assays and clinically relevant infection scenarios. A substantial proportion of the current literature relies on planktonic bacterial cultures and short-term antimicrobial assays, which fail to capture the spatial heterogeneity, metabolic gradients, and microbial diversity characteristic of chronic wounds or polymicrobial infections. Addressing this limitation requires broader adoption of advanced experimental models. Ex vivo human skin systems represent a particularly promising platform because they preserve the structural integrity of the epidermal barrier and provide a realistic biochemical microenvironment for evaluating peptide penetration, retention, and local toxicity. When combined with well-controlled in vivo infection models, these systems offer an opportunity to improve translational predictability while reducing the dependence on extensive animal experimentation.

Looking forward, several strategic directions are likely to shape the next stage of AMP development. First, rational integration of peptide engineering with advanced delivery systems will be essential to maximize antimicrobial efficacy while minimizing cytotoxicity. Second, experimental pipelines must increasingly incorporate models that reproduce biofilm-associated infections and impaired wound healing conditions, which represent the most relevant clinical settings for topical AMP therapies. Third, standardization of experimental protocols—including infection models, biofilm assays, and wound healing metrics—will be critical to enable meaningful comparison between studies and reduce inconsistencies across the literature. Finally, issues related to manufacturing scalability, formulation stability, and regulatory reproducibility should be addressed early in development to avoid the frequent translational bottlenecks observed for peptide therapeutics.

In this context, the present review highlights an emerging paradigm in AMP research: antimicrobial peptides should not be considered solely as molecular antibacterial agents but as functional components of integrated therapeutic platforms. Their greatest clinical potential may lie in localized delivery systems capable of simultaneously controlling infection, modulating inflammation, and promoting tissue regeneration. By focusing on the interplay between peptide chemistry, formulation design, and biologically relevant evaluation models, this work aims to provide a perspective that complements existing AMP reviews and emphasizes the importance of context-driven strategies for the successful clinical translation of peptide-based antimicrobials.

## 5. Conclusions

Antimicrobial peptides exhibit a broad range of biological activities that extend beyond direct bacterial killing. As outlined in this review, their mechanisms of action include membrane destabilization, interference with cell wall biosynthesis, disruption of biofilms, and modulation of local immune responses. These combined effects make AMPs particularly suited for topical treatment strategies, where microbial persistence, inflammation, and tissue damage are tightly interconnected. The studies discussed here consistently show that formulation plays a decisive role in determining peptide performance. Incorporation into hydrogels, polymeric films, nanofibrous dressings, and composite matrices improves peptide stability, reduces cytotoxicity, and enables sustained local activity. In this context, the delivery system is not a secondary consideration but a core component of therapeutic efficacy. Advances in peptide modification and conjugation have further expanded the functional space of AMPs, allowing fine control over selectivity, toxicity, and biological stability. When combined with robust in vivo and ex vivo evaluation, these strategies provide a realistic framework for assessing both antimicrobial activity and tissue compatibility. Overall, antimicrobial peptides should be viewed not as direct substitutes for conventional antibiotics, but as complementary agents capable of addressing infection, biofilm persistence, and local tissue responses in parallel. Continued progress in this field will depend on rational design choices that integrate peptide chemistry, formulation engineering, and biologically relevant testing, ultimately supporting the development of effective and safe topical anti-infective therapies.

## Figures and Tables

**Table 1:** Topical formulations of antimicrobial peptides: materials, experimental evaluation, and key outcomes.

Topical Formulation	AMP	Matrix Material	Experimental Evaluation	Key Results	Reference
Hydrogel	AMC-109	Polymeric gel + cotton dressing	In vitro (CFU count, inhibition zone); in vivo (*S. aureus*, MRSA)	99% reduction in infection; dose-dependent activity superior to commercial topical agents	[69]
Hydrogel	LL-37	Chitosan (2.5% *w*/*v*)	In vitro (CFU count); in vivo (mouse model)	Reduced bacterial burden; enhanced vascularization and re-epithelialization	[63]
Nanofibrous dressing	NP10	Chitosan + PEO (CS/PEO)	In vitro and in vivo (*S. aureus* and *E. coli*)	Inhibition of bacterial growth; accelerated wound healing	[65]
Nanofibrous dressing	Pexiganan + Tiger 17	PVA + cellulose acetate	In vitro (CFU count); hemostasis; cytotoxicity	Inhibition of *S. aureus* and *P. aeruginosa*; hemostatic effect; high biocompatibility	[68]
Polymeric film	ε-Poly-L-lysine (ε-PLL)	Chitosan + PVA	In vitro (agar diffusion assay, cytotoxicity)	Antibacterial activity against *S. aureus* and *E. coli*; high biocompatibility	[50]
Chitosan nanoparticles	Temporin B	Chitosan nanoparticles	In vitro (*S. epidermidis*)	Prolonged bactericidal activity against *S. epidermidis*	[76]
Polymeric film/hydrogel	IP-1 and W379	GelMA + MXene	Infected diabetic wounds (*S. aureus*)	AMP–nanoparticle synergy promoted rapid wound healing	[77]
Hydrogel/polymeric film	RRP9W4N	Quaternized chitosan (QCS) + GelMA	*S. aureus*, MRSA, *P. aeruginosa*, *E. coli*; in vivo (rat model)	High stability and prolonged antiseptic activity; effective in vivo performance	[78]
Dressing	HA/ALG/SF nanofiber AMP (HA-AMP)	PVA–alginate (PVA–ALG)	Cutaneous infections in rats	Controlled release; promoted angiogenesis and rapid wound healing	[79]

**Table 2:** Examples of wound healing active nanoformulations based in nanostructured systems for antimicrobial peptides encapsulation/delivery.

AMP Nanoformulation	Peptide	Structure Information	Microorganism	Highlights	Ref.
Omiganan lipossomal gel	Omiganan	Amino acid sequence: ILRWPWWPWRRK-NH2	*Staphylococcus aureus*	The liposomal system provided controlled peptide release, improved skin permeation, and exhibited anti-inflammatory activity by reducing pro-inflammatory cytokine levels in atopic dermatitis and psoriasis lesions of mouse models.	[83]
P1-AMP liposome-loaded hydrogel	P1-AMP	Amino acid sequence: VVVNVLVKVLPPPVV	*Staphylococcus aureus* and MRSA *Staphylococcus aureus*	It exhibits strong antimicrobial and wound healing activity by enhancing the permeability of the P1-AMP in the skin, in addition to increasing the stability of the peptide when stored.	[84]
Azithromycin and nisin-loaded liposome formulations	Nisin	Polycyclic structure	*Staphylococcus aureus* and biofilm of *S. aureus*	The mixing of lipids in the liposome structure with cellular lipids in the stratum corneum enhanced skin moisturization and the penetration of nisin, in addition to present superior antimicrobial/antibiofilm activities and biocompatibilities when compared to azitromicin-loaded liposome.	[85]
Nisin-loaded PLGA nanoparticles				Nisin-loaded PLGA nanoparticles are a more suitable drug delivery system compared to nisin alone, showing better antimicrobial activity against free bacteria and biofilm formations in comparison to the free peptide.	[86]
LL37 nanostructured lipid carriers	LL37	Amino acid sequence: LLGDFFRKSKEKI GKEFKRIVQRIK DFLRNLVPRTES	*Escherichia coli*	The LL37 NLC significantly improved healing compared to the same concentration of the LL37 solution in terms of wound closure, re-epithelization grade and restoration of the inflammatory process.	[87,88]
LL37 + serpin A1 solid lipid nanoparticles			*Staphylococcus aureus* and *Escherichia coli*	The sinergystic effect of LL37 peptide with serpin A1, an elastase inhibitor with wound healing properties, when incorporated in SLNs showed to enhance antimicrobial and antinflammatory activity while accelerating the wound healing process.	[89]
LL37 chitosan hydrogel			*Staphylococcus aureus*	In a mouse model of wound injuries, the LL37 CS hydrogel significantly reduced the area of pressure ulcers and enhanced epithelial thickness as well as the density of newly formed capillaries when compared with free LL37.	[63]
LL37-loaded chitosan nanoparticles			Methicillin-resistant *Staphylococcus aureus*	LL37-loaded CS nanoparticles prolonged the antibacterial activity of the AMP by increasing its half-life, in addition to its 68% biofilm formation inhibition, compared to the analyses of LL37 alone.	[90]
DPK-060 dendritic nanogel embedded into poloxamer gel	DPK-060	Amino acid sequence: GKHKNKGKKNGKHNGWKWWW	*Staphylococcus aureus*	DPK-060 dendritic nanogel improved the AMP antimicrobial efficiency in inhibiting the growth of *S. aureus*, observed in vitro, ex vivo and in vivo anti-infection tests, presenting a slower release in comparison with free peptide.	[91]
Nucleic acid L12 nanogel	L12	Peptide analog derived from an *Enterococcus faecium* enterocin	*Staphylococcus aureus*	Reduction in clinical symptons and microbial burden of the bacteria in models of *S. aureus* infectious bacterial keratitis.	[92]

**Table 3:** Intramolecular modifications of antimicrobial peptides and their impact on antimicrobial activity and biological performance.

Modification	Peptide	Structure/Sequence	Antimicrobial Activity	Objective	Observed Impact	Ref.
Phosphorylation	Salivaricin 10	—	Gram-positive bacteria: 0.125–64 µg/mL Gram-negative bacteria: 32–64 µg/mL	To investigate post-translational modifications and associated biological functions of the lantipeptide	Phosphorylation was essential for antimicrobial, antibiofilm, and immunoregulatory activity	[102]
	W3BipY8-P	GL(Bip)KRLKY(P)LL-NH_2_	*S. aureus* ATCC 25923: 8 µM *B. subtilis* ATCC 23857: 8 µM *E. coli* ATCC 25922: 8 µM *P. aeruginosa* ATCC 27853: 16 µM *K. pneumoniae* ATCC 700603: 8 µM *A. baumannii* ATCC 19606: 4 µM	To evaluate phosphorylation as a strategy to reduce toxicity and hemolysis	19-fold reduction in hemolytic toxicity and 3.3-fold decrease in cytotoxicity while preserving antimicrobial activity	[120]
Glycosylation	g-LL-III	VNWKKILGKIIKVVK-NH_2_	*E. coli*, *P. aeruginosa*, *A. baumannii*, *K. pneumoniae*, MRSA: 3.12 µM *S. aureus*, *B. subtilis*, *B. globigii*: 1.56 µM	To enhance resistance to proteolytic degradation	Improved protease stability without significant loss of antimicrobial potency	[105]
	[βGlc-T9,K7]Indolicidin	ILPWKWPWWPWRR-NH_2_	*P. aeruginosa*, *S. aureus*, *E. coli*: 3 µM *S. typhi*: 2 µM	To reduce hemolytic activity and hydrophobicity	Decreased cytotoxicity and increased solubility	[121]
	g-G3	GIIKKIIKKIIKKIS-NH_2_-Glc	*P. aeruginosa* and *S. aureus*: 16 µM	To assess C-terminal glycosylation effects	Slightly reduced planktonic activity but enhanced antibiofilm activity, increased hydrophilicity, and reduced hemolysis	[122]
Acetylation	L163-Ac	Ac-FLPLIGGLLKGLL-NH_2_	*Streptococcus* Sc181: 1.95 ± 0.02 µM *L. monocytogenes*: 1.95 ± 0.02 µM *Enterococcus* E1478F: 3.91 ± 0.02 µM *S. aureus*: 3.91 ± 0.05 µM	To improve stability against proteolysis, temperature, and pH	Enhanced stability and increased antibacterial activity, despite reduced antifungal activity	[123]
	Ac-L1A	Ac-IDGLKAIWKKVADLLKNT-NH_2_	Lytic activity in model membranes (MIC not determined)	To neutralize *N*-terminal positive charge and enhance membrane lysis	Increased membrane disruption and deeper bilayer penetration	[114]
Methylation	C10:0-A2(6-NMeLys)	C10:0-IKQVK(NMe-K)LFKK-NH_2_	*E. coli* ATCC 35218: 1.4 µM *P. aeruginosa* ATCC 27853: 1.44 µM *S. aureus* RM-SA, *E. faecalis* ATCC 29212: 5.6 µM *C. tropicalis* DBFIQ3: 89.7 µM	To optimize stability and therapeutic efficacy	Reduced hemolytic toxicity, though overall antimicrobial activity decreased; improved activity against *C. albicans*	[124]
	*N*-methylated Gramicidin S (NMe-GS)	—	MDRSA: 3.6 µM VRSA: 3.6 µM MRSE: 1.8 µM *E. coli*: 28 µM *K. pneumoniae*: 3.6 µM *Salmonella*: 29 µM	To dissociate antimicrobial and hemolytic activities	Reduced hemolysis without significant loss of antibacterial efficacy	[116]
Cyclization	Magainin 2 (analog 2)	H-S_5_IKKS_5_LKSAKKFVKAFK-NH_2_	*S. aureus*: 3.125 µM *E. coli*: 1.56 µM *P. aeruginosa*: 1.56 µM MDR *P. aeruginosa*: 1.56 µM	To stabilize structure via peptide stapling	Markedly increased antibacterial activity and reduced hemolysis	[118]
	CE-05	cyclo-(RRRRRRWWWWVVVV)	*P. aeruginosa*: 6.1 µM *A. baumannii*: 1.1 µM *K. pneumoniae*: 6.7 µM *E. coli*: 2.5 µM *Enterobacter*: 3.2 µM *Enterococcus*: 0.6 µM *S. aureus*: 1.0 µM	To enhance bactericidal efficacy, proteolytic stability, and reduce toxicity	Superior antimicrobial performance and partial resistance to enzymatic degradation	[119]

**Table 4:** Extramolecular conjugation strategies applied to antimicrobial peptides and their biological impact.

Strategy	Peptide	Structure/Sequence	Antimicrobial Activity	Objective	Observed Impact	Ref.
PEGylation	SAAP-148 (analogs)	Ac-LKRVWKRVFKLLKRYWRQLKKPVR-NH_2_ (PEG conjugated at different residues)	Expressed as LC_99_._9_:*S. aureus* (plasma) LUH14960: 25.6–51.2 µM MRSA (plasma) LUH14616: 12.8–51.2 µM	To develop PEGylated analogs of different lengths to reduce cytotoxicity and extend circulation half-life	Retained antibacterial activity with reduced hemolysis and enhanced immunomodulatory effects compared to the parent peptide	[126]
	PEGylated N6 (N6-COOH-miniPEG)	GFAWNVCVYRNGVRVCHRRAN-miniPEG	Gram-negative bacteria: 3.05–24.42 µM Gram-positive bacteria: 64 to >128 µM	To improve N6 stability against trypsin without compromising antibacterial activity	Significantly increased proteolytic stability and preserved anti-inflammatory activity, with only a slight reduction in in vivo efficacy	[135]
Lipidation	KR12-NH_2_ (IV)	C8-KR12-NH_2_	*E. faecium*: 1 µM *K. pneumoniae*, *A. baumannii*, *P. aeruginosa*, *K. aerogenes*: 2 µM	To evaluate the effect of *N*-terminal lipophilic modifications	Analog IV exhibited the lowest MIC among tested peptides and eradicated *S. aureus* biofilms at low concentrations	[127]
	Peptide C (lipidated 1B)	H-Phe(4-NHCO(CH_2_)_3_CH_3_)-Val-Pro-Trp-Phe-Ser-Lys-Phe-DLeu-DLys-Arg-Ile-Leu-NH_2_	MIC 6.25–25 µM against carbapenemase-producing *K. pneumoniae* (KPC)MIC 25 µM against metallo-β-lactamase-producing strains (MBL)	To assess activity against carbapenem-resistant *K. pneumoniae*	Higher activity than peptide 1B; no cytotoxicity observed in human keratinocytes or erythrocytes at the highest concentration tested	[136]
Antibiotic–peptide	CPFx-1 and CPFx-2	AMP conjugated to ciprofloxacin via ester bonds	*E. coli*: 1.56 µM *S. aureus*: 3.125 µM	To enhance efficacy and reduce resistance development through a synergistic strategy	Increased broad-spectrum bactericidal activity; demonstrated in vivo efficacy and pro-healing effects	[128]
	Vancomycin–PMEN	Covalent conjugation of vancomycin to polymyxin E nonapeptide (PMEN)	Gram-positive bacteria: ≤2 to ≥16 µg/mL Gram-negative bacteria: ≥2- to ≥8/16-fold activity increase	To generate “vancomyxins” with expanded Gram-negative activity	Maintained or enhanced Gram-positive activity, improved Gram-negative efficacy, and reduced renal toxicity compared to polymyxin	[137]
Siderophore–peptide	P1-DFP	K(RW)_3_-DFP-COOH	*P. aeruginosa* (siderophore-producing strains): 2 µM *P. aeruginosa* (pyoverdine/pyochelin-deficient strains): 0.25–1 µM	To overcome the Gram-negative envelope barrier using a “Trojan horse” strategy	Enhanced antimicrobial activity, demonstrating efficient periplasmic delivery of K(RW)_3_	[133]
	Pep-cyc3	—	*K. pneumoniae* in iron(III)-supplemented medium: 6.25 µM	To investigate antimicrobial mechanism using fluorescence, CD, and NMR analyses	Higher antimicrobial activity than the precursor peptide due to optimized structure and chelating capacity	[138]
	MccE492m	GETDPNTQLLNDLGNNMAWG AALGAPGGLGSAALGAAGGALQTVGQGLIDHGPVNVPIPVLIGPSWNGSSSGYNSATSSSG + siderophore	*E. coli* B: 0.04 µM *E. coli* F: 0.08 µM *E. coli* ML35p: 0.08 µM *S. enteritidis*: 0.15 µM *E. cloacae*: 0.60 µM *K. pneumoniae*: 2.50 µM	To isolate and characterize the post-translational siderophore modification and assess antibacterial activity	Enhanced potency compared to MccE492; inactive against non-Enterobacteriaceae and Gram-positive bacteria	[139]
Antibody–peptide	VSX-1	—	MIC values not reported for the conjugate (components tested separately)	To combine peptide antimicrobial activity with antibody specificity against *P. aeruginosa*	Improved therapeutic performance compared to individual components; promising in vivo results	[140]
	ABC1 conjugate	Cyclic macro-peptide linked to a monoclonal antibody	Viability assays performed; MIC not reported	To integrate antibody targeting with peptide antibacterial potency	Retained activity, primarily against *E. coli*, at nanomolar concentrations without detectable hemolysis	[141]

**Table 5:** In vivo studies evaluating antimicrobial peptides using different animal models and therapeutic approaches.

AMP	Animal Model	Therapeutic Approach	References
Nisin	Mouse	*Staphylococcus aureus* infection	[159]
APO	Mouse	*Acinetobacter baumannii* infection	[160]
LL-37	Diabetic mouse	*Staphylococcus epidermidis* and *Pseudomonas aeruginosa* infection	[161]
NP10	Rat	*Staphylococcus aureus* infection	[65]
hBD-3	Mice	Wound healing evaluation	[157]
DPK-060	Mouse	*Staphylococcus aureus* infection	[96]
RRP9W4N	Mice	*Staphylococcus aureus* infection	[78]
ACP5	Guinea pig	*Trichophyton mentagrophytes* infection	[158]

## Data Availability

No new data were created or analyzed in this study. Data sharing is not applicable to this article.

## Abbreviations

ALG	Alginate
AMPs	Antimicrobial peptides
AMR	Antimicrobial resistance
ATCC	American Type Culture Collection
CA	Cellulose acetate
CD	Circular dichroism spectroscopy
CDI	Carbonyldiimidazole
CFU	Colony-forming unit
CS	Chitosan
DNA	Deoxyribonucleic acid
EPS	Extracellular polymeric substances
FGF	Fibroblast growth factor
FTIR	Fourier transform infrared spectroscopy
GelMA	Gelatin methacryloyl
GMP	Good Manufacturing Practice
HA	Hyaluronic acid
hBD-3	Human β-defensin-3
IL	Interleukin (e.g., IL-8, IL-6, IL-1α, IL-1β)
KPC	Klebsiella pneumoniae carbapenemase
LPS	Lipopolysaccharide
LTA	Lipoteichoic acid
MAPK	Mitogen-activated protein kinase
MBL	Metallo-β-lactamase
MCP-1	Monocyte chemoattractant protein-1
MDRSA	Multidrug-resistant Staphylococcus aureus
MIC	Minimum inhibitory concentration
MRSA	Methicillin-resistant Staphylococcus aureus
MRSE	Methicillin-resistant Staphylococcus epidermidis
MXene	Two-dimensional transition metal carbides and nitride
NAG	N-acetylglucosamine
NAM	N-acetylmuramic acid
NF-κB	Nuclear factor-κB
NLCs	Nanostructured lipid carriers
NMR	Nuclear magnetic resonance spectroscopy
O/W	Oil-in-water
PBS	Phosphate-buffered saline
PDGF	Platelet-derived growth factor
PEG	Poly(ethylene glycol)
PEO	Poly(ethylene oxide)
PLA	Poly(lactic acid)
PLGA	Poly(lactide-co-glycolide)
PNs	Polymeric nanoparticles
PU	Polyurethane
PVA	Poly(vinyl alcohol)
QCS	Quaternized chitosan
RNA	Ribonucleic acid
SF	Silk fibroin
SLNs	Solid lipid nanoparticles
VEGF	Vascular endothelial growth factor
VRSA	Vancomycin-resistant Staphylococcus aureus
W/O	Water-in-oil
WVTR	Water vapor transmission rate
ε-PLL	ε-poly-L-lysine

## References

[1] Ho C.S., Wong C.T.H., Aung T.T., Lakshminarayanan R., Mehta J.S., Rauz S., McNally A., Kintses B., Peacock S.J., de la Fuente-Nunez C. (2025). Antimicrobial Resistance: A Concise Update. Lancet Microbe.

[2] Brink A.J., Richards G.A. (2020). The Role of Multidrug and Extensive-Drug Resistant Gam-Negative Bacteria in Skin and Soft Tissue Infections. Curr. Opin. Infect. Dis..

[3] Cardona S.T., Rahman A.S.M.Z., Novomisky Nechcoff J. (2025). Innovative Perspectives on the Discovery of Small Molecule Antibiotics. npj Antimicrob. Resist..

[4] Talha M., Roque-Borda C.A. (2026). Enhanced Antibacterial Activity of Antimicrobial Peptide–Antibiotic Combinations Against Multidrug-Resistant Bacteria. FEMS Microbes.

[5] Roque-Borda C.A., Vishwakarma S.K., Ramirez Delgado O.J., de Souza Rodrigues H.L., Primo L.M.D., Campos I.C., de Lima T.S., Perdigão J., Pavan F.R. (2025). Peptide-Based Strategies Against *Mycobacterium tuberculosis* Covering Immunomodulation, Vaccines, Synergistic Therapy, and Nanodelivery. Pharmaceuticals.

[6] Carnero Canales C.S., Roque-Borda C.A., Cazorla J.I.M., Cazorla R.M.M., Apaza U.J.P., Silva V.d.J., Primo L.M.D.G., Martínez-Morales M.J., Miguel Sábio R., Santos H.A. (2025). Forging a New Frontier: Antimicrobial Peptides and Nanotechnology Converging to Conquer Gastrointestinal Pathogens. Small.

[7] Polinário G., Primo L.M.D.G., Rosa M.A.B.C., Dett F.H.M., Barbugli P.A., Roque-Borda C.A., Pavan F.R. (2023). Antimicrobial Peptides as Drugs with Double Response against *Mycobacterium tuberculosis* Coinfections in Lung Cancer. Front. Microbiol..

[8] Torres M.D.T., Cesaro A., de la Fuente-Nunez C. (2025). Peptides from Non-Immune Proteins Target Infections through Antimicrobial and Immunomodulatory Properties. Trends Biotechnol..

[9] Roque-Borda C.A., Zhang Q., Nguyen T.P.T., Nguyen T.T.H., Medhi H., Rodrigues H.L.d.S., Canales Carnero C.S., Sutherland D., Helmy N.M., Araveti P.B. (2026). Synergistic Combinations of Antimicrobial Peptides and Conventional Antibiotics: A Strategy to Delay Resistance Emergence in World Health Organization Priority Bacteria. Pharmacol. Rev..

[10] Gamal H., Roque-Borda C.A., de la Torre B.G., Albericio F. (2026). Structure Function and Design of Polymyxins to Enable Safer and More Potent Anti Gram Negative Agents. Eur. J. Med. Chem..

[11] Moradi S.V., Hussein W.M., Varamini P., Simerska P., Toth I. (2016). Glycosylation, an Effective Synthetic Strategy to Improve the Bioavailability of Therapeutic Peptides. Chem. Sci..

[12] Tsylents U., Burmistrz M., Wojciechowska M., Stępień J., Maj P., Trylska J. (2024). Iron Uptake Pathway of *Escherichia coli* as an Entry Route for Peptide Nucleic Acids Conjugated with a Siderophore Mimic. Front. Microbiol..

[13] Sun H., Hong Y., Xi Y., Zou Y., Gao J., Du J. (2018). Synthesis, Self-Assembly, and Biomedical Applications of Antimicrobial Peptide–Polymer Conjugates. Biomacromolecules.

[14] Li W., Separovic F., O’Brien-Simpson N.M., Wade J.D. (2021). Chemically Modified and Conjugated Antimicrobial Peptides against Superbugs. Chem. Soc. Rev..

[15] Baral K.C., Choi K.Y. (2025). Barriers and Strategies for Oral Peptide and Protein Therapeutics Delivery: Update on Clinical Advances. Pharmaceutics.

[16] Mu J., Vong E., Carmali S. (2025). Artificial Lipidation of Proteins and Peptides: From Mechanism to Clinical Applications. FEBS J..

[17] Zheng S., Tu Y., Li B., Qu G., Li A., Peng X., Li S., Shao C. (2025). Antimicrobial Peptide Biological Activity, Delivery Systems and Clinical Translation Status and Challenges. J. Transl. Med..

[18] Lin L., Chi J., Yan Y., Luo R., Feng X., Zheng Y., Xian D., Li X., Quan G., Liu D. (2021). Membrane-Disruptive Peptides/Peptidomimetics-Based Therapeutics: Promising Systems to Combat Bacteria and Cancer in the Drug-Resistant Era. Acta Pharm. Sin. B.

[19] Steinbuch K.B., Fridman M. (2016). Mechanisms of Resistance to Membrane-Disrupting Antibiotics in Gram-Positive and Gram-Negative Bacteria. Medchemcomm.

[20] Rohde M. (2019). The Gram-Positive Bacterial Cell Wall. Microbiol. Spectr..

[21] Cao Y., Lin H. (2021). Characterization and Function of Membrane Vesicles in Gram-Positive Bacteria. Appl. Microbiol. Biotechnol..

[22] Van Bambeke F., Mingeot-Leclercq M.P., Struelens M.J., Tulkens P.M. (2008). The Bacterial Envelope as a Target for Novel Anti-MRSA Antibiotics. Trends Pharmacol. Sci..

[23] Malanovic N., Lohner K. (2016). Gram-Positive Bacterial Cell Envelopes: The Impact on the Activity of Antimicrobial Peptides. Biochim. Biophys. Acta (BBA)—Biomembr..

[24] Hasper H.E., Kramer N.E., Smith J.L., Hillman J.D., Zachariah C., Kuipers O.P., De Kruijff B., Breukink E. (2006). An Alternative Bactericidal Mechanism of Action for Lantibiotic Peptides That Target Lipid II. Science.

[25] Hsu S.T.D., Breukink E., Tischenko E., Lutters M.A.G., De Kruijff B., Kaptein R., Bonvin A.M.J.J., Van Nuland N.A.J. (2004). The Nisin-Lipid II Complex Reveals a Pyrophosphate Cage That Provides a Blueprint for Novel Antibiotics. Nat. Struct. Mol. Biol..

[26] Malanovic N., Lohner K. (2016). Antimicrobial Peptides Targeting Gram-Positive Bacteria. Pharmaceuticals.

[27] Li Z., Zhang S., Zhang J., Liu M., Liu Z. (2009). Vitellogenin Is a Cidal Factor Capable of Killing Bacteria via Interaction with Lipopolysaccharide and Lipoteichoic Acid. Mol. Immunol..

[28] Ruhal R., Kataria R. (2021). Biofilm Patterns in Gram-Positive and Gram-Negative Bacteria. Microbiol. Res..

[29] Abee T., Kovács Á.T., Kuipers O.P., van der Veen S. (2011). Biofilm Formation and Dispersal in Gram-Positive Bacteria. Curr. Opin. Biotechnol..

[30] Arciola C.R., Campoccia D., Montanaro L. (2018). Implant Infections: Adhesion, Biofilm Formation and Immune Evasion. Nat. Rev. Microbiol..

[31] Muhammad M.H., Idris A.L., Fan X., Guo Y., Yu Y., Jin X., Qiu J., Guan X., Huang T. (2020). Beyond Risk: Bacterial Biofilms and Their Regulating Approaches. Front. Microbiol..

[32] Yasir M., Willcox M.D.P., Dutta D. (2018). Action of Antimicrobial Peptides against Bacterial Biofilms. Materials.

[33] Segev-Zarko L., Saar-Dover R., Brumfeld V., Mangoni M.L., Shai Y. (2015). Mechanisms of Biofilm Inhibition and Degradation by Antimicrobial Peptides. Biochem. J..

[34] Li Y., Li S., Yang K., Guo R., Zhu X., Shi Y., Huang A. (2022). Antibiofilm Mechanism of a Novel Milk-derived Antimicrobial Peptide against *Staphylococcus aureus* by Downregulating Agr Quorum Sensing System. J. Appl. Microbiol..

[35] Duarte-Mata D.I., Salinas-Carmona M.C. (2023). Antimicrobial Peptides’ Immune Modulation Role in Intracellular Bacterial Infection. Front. Immunol..

[36] Auvynet C., Rosenstein Y. (2009). Multifunctional Host Defense Peptides: Antimicrobial Peptides, the Small yet Big Players in Innate and Adaptive Immunity. FEBS J..

[37] Brunetti J., Roscia G., Lampronti I., Gambari R., Quercini L., Falciani C., Bracci L., Pini A. (2016). Immunomodulatory and Anti-Inflammatory Activity in Vitro and in Vivo of a Novel Antimicrobial Candidate. J. Biol. Chem..

[38] Pfalzgraff A., Heinbockel L., Su Q., Gutsmann T., Brandenburg K., Weindl G. (2016). Synthetic Antimicrobial and LPS-Neutralising Peptides Suppress Inflammatory and Immune Responses in Skin Cells and Promote Keratinocyte Migration. Sci. Rep..

[39] Thapa R.K., Diep D.B., Tønnesen H.H. (2020). Topical Antimicrobial Peptide Formulations for Wound Healing: Current Developments and Future Prospects. Acta Biomater..

[40] Lopes A.I., Pintado M.M., Tavaria F.K. (2024). Plant-Based Films and Hydrogels for Wound Healing. Microorganisms.

[41] Zhang W., Liu L., Cheng H., Zhu J., Li X., Ye S., Li X. (2023). Hydrogel-Based Dressings Designed to Facilitate Wound Healing. Mater. Adv..

[42] Yu P., Wei L., Yang Z., Liu X., Ma H., Zhao J., Liu L., Wang L., Chen R., Cheng Y. (2024). Hydrogel Wound Dressings Accelerating Healing Process of Wounds in Movable Parts. Int. J. Mol. Sci..

[43] Gounden V., Singh M. (2024). Hydrogels and Wound Healing: Current and Future Prospects. Gels.

[44] Shen Z., Zhang C., Wang T., Xu J. (2023). Advances in Functional Hydrogel Wound Dressings: A Review. Polymers.

[45] Oliveira R., Almeida I.F. (2023). Patient-Centric Design of Topical Dermatological Medicines. Pharmaceuticals.

[46] Esposito T., Mencherini T., Sansone F., Auriemma G., Gazzerro P., Puca R.V., Iandoli R., Aquino R.P. (2021). Development, Characterization, and Clinical Investigation of a New Topical Emulsion System Containing a Castanea Sativa Spiny Burs Active Extract. Pharmaceutics.

[47] Badruddoza A.Z.M., Yeoh T., Shah J.C., Walsh T. (2023). Assessing and Predicting Physical Stability of Emulsion-Based Topical Semisolid Products: A Review. J. Pharm. Sci..

[48] Savencu I., Iurian S., Porfire A., Bogdan C., Tomuță I. (2021). Review of Advances in Polymeric Wound Dressing Films. React. Funct. Polym..

[49] Borbolla-Jiménez F.V., Peña-Corona S.I., Farah S.J., Pineda-Pérez E., Romero-Montero A., Alberto S., Magaña J.J., Leyva-González G. (2023). Films for Wound Healing Fabricated Using a Solvent Casting Technique. Pharmaceutics.

[50] Gonçalves M.M., Carneiro J., Justus B., Espinoza J.T., Budel J.M., Farago P.V., de Paula J.P. (2020). Preparation and Characterization of a Novel Antimicrobial Film Dressing for Wound Healing Application. Braz. J. Pharm. Sci..

[51] Ibrahim M.A., Nasrallah D.A., El N.M., Omar S. (2024). Selenium Loaded Sodium Alginate / Polyvinyl Alcohol Nanocomposite Film as Wound Dressing: Structural, Optical, Mechanical, Antimicrobial Properties and Biocompatibility. Appl. Phys. A.

[52] Buriti B.M.A.D.B., Figueiredo P.L.B., Passos M.F., Kelly J., Silva R. (2024). Polymer-Based Wound Dressings Loaded with Essential Oil for the Treatment of Wounds: A Review. Pharmaceuticals.

[53] Roque-Borda C.A., Antunes B.F., Toledo Borgues A.B., Costa de Pontes J.T., Meneguin A.B., Chorilli M., Trovatti E., Teixeira S.R., Pavan F.R., Vicente E.F. (2022). Conjugation of Ctx(Ile^21^)-Ha Antimicrobial Peptides to Chitosan Ultrathin Films by N-Acetylcysteine Improves Peptide Physicochemical Properties and Enhances Biological Activity. ACS Omega.

[54] Lu X., Zhou L., Song W. (2024). Recent Progress of Electrospun Nanofiber Dressing in the Promotion of Wound Healing. Polymers.

[55] Yan B., Zhang Y., Li Z., Zhou P., Mao Y. (2022). Electrospun Nanofibrous Membrane for Biomedical Application. SN Appl. Sci..

[56] Holloway S., Harding K.G. (2022). Wound Dressings. Surgery.

[57] Hodge J.G., Zamierowski D.S., Robinson J.L., Mellott A.J. (2022). Evaluating Polymeric Biomaterials to Improve Next Generation Wound Dressing Design.

[58] Soylu Z., Oktay B., Erarslan A., Ahlatcıoğlu E. (2025). Multifunctional Polymeric Wound Dressings.

[59] Manivannan R.K., Sharma N., Kumar V., Jayaraj I., Vimal S. (2024). A Comprehensive Review on Natural Macromolecular Biopolymers for Biomedical Applications: Recent Advancements, Current Challenges, and Future Outlooks. Carbohydr. Polym. Technol. Appl..

[60] Pfalzgraff A., Brandenburg K., Weindl G. (2018). Antimicrobial Peptides and Their Therapeutic Potential for Bacterial Skin Infections and Wounds. Front. Pharmacol..

[61] Agier J., Brzezińska-Błaszczyk E., Zelechowska P., Wiktorska M., Pietrzak J., Rózalska S. (2018). Cathelicidin LL-37 Affects Surface and Intracellular Toll-like Receptor Expression in Tissue Mast Cells. J. Immunol. Res..

[62] Vignesh S., Sivashanmugam A., Annapoorna M., Janarthanan R., Iyer S., Nair S.V., Jayakumar R. (2018). Injectable Deferoxamine Nanoparticles Loaded Chitosan-Hyaluronic Acid Coacervate Hydrogel for Therapeutic Angiogenesis. Colloids Surf. B Biointerfaces.

[63] Yang X., Guo J.L., Han J., Si R.J., Liu P.P., Zhang Z.R., Wang A.M., Zhang J. (2020). Chitosan Hydrogel Encapsulated with LL-37 Peptide Promotes Deep Tissue Injury Healing in a Mouse Model. Mil. Med. Res..

[64] Abrigo M., McArthur S.L., Kingshott P. (2014). Electrospun Nanofibers as Dressings for Chronic Wound Care: Advances, Challenges, and Future Prospects. Macromol. Biosci..

[65] Yu L., Dou S., Ma J., Gong Q., Zhang M., Zhang X., Li M., Zhang W. (2021). An Antimicrobial Peptide-Loaded Chitosan/Polyethylene Oxide Nanofibrous Membrane Fabricated by Electrospinning Technology. Front. Mater..

[66] Mangoni M.L., Mcdermott A.M., Zasloff M. (2016). Antimicrobial Peptides and Wound Healing: Biological and Therapeutic Considerations. Exp. Dermatol..

[67] Felgueiras H.P., Amorim M.T.P. (2017). Functionalization of Electrospun Polymeric Wound Dressings with Antimicrobial Peptides. Colloids Surf. B Biointerfaces.

[68] Teixeira M.A., Antunes J.C., Seabra C.L., Tohidi S.D., Reis S., Amorim M.T.P., Felgueiras H.P. (2022). Tiger 17 and Pexiganan as Antimicrobial and Hemostatic Boosters of Cellulose Acetate-Containing Poly(Vinyl Alcohol) Electrospun Mats for Potential Wound Care Purposes. Int. J. Biol. Macromol..

[69] Håkansson J., Cavanagh J.P., Stensen W., Mortensen B., Svendsen J.-S., Svenson J. (2021). In Vitro and in Vivo Antibacterial Properties of Peptide AMC-109 Impregnated Wound Dressings and Gels. J. Antibiot..

[70] Gomes D., Santos R., Soares R.S., Reis S., Carvalho S., Rego P., Peleteiro M.C., Tavares L., Oliveira M. (2020). Pexiganan in Combination with Nisin to Control Polymicrobial Diabetic Foot Infections. Antibiotics.

[71] Song Y., Lu Y., Wang L., Yang H., Zhang K., Lai R. (2009). Purification, Characterization and Cloning of Two Novel Tigerinin-like Peptides from Skin Secretions of *Fejervarya cancrivora*. Peptides.

[72] Amariei G., Kokol V., Boltes K., Letón P., Rosal R. (2018). Incorporation of Antimicrobial Peptides on Electrospun Nanofibres for Biomedical Applications. RSC Adv..

[73] Zhang X., Wang Y., Gao Z., Mao X., Cheng J., Huang L., Tang J. (2024). Advances in Wound Dressing Based on Electrospinning Nanofibers. J. Appl. Polym. Sci..

[74] Teixeira M.A., Murthy N.S., Ferreira D.P., Felgueiras H.P. (2024). Assessment of Linear and Cyclic Peptides’ Immobilization onto Cross-Linked, Poly(Vinyl Alcohol)/Cellulose Nanocrystal Nanofibers Electrospun over Quartz Crystal Microbalances with Dissipation Sensors. Langmuir.

[75] Wang Y., Zhang Y., Su R., Wang Y., Qi W. (2024). Antimicrobial Therapy Based on Self-Assembling Peptides. J. Mater. Chem. B.

[76] Piras A.M., Maisetta G., Sandreschi S., Gazzarri M., Bartoli C., Grassi L., Esin S., Chiellini F., Batoni G. (2015). Chitosan Nanoparticles Loaded with the Antimicrobial Peptide Temporin B Exert a Long-Term Antibacterial Activity in Vitro against Clinical Isolates of Staphylococcus Epidermidis. Front. Microbiol..

[77] Liang C., Wang H., Lin Z., Zhang C., Liu G., Hu Y. (2023). Augmented Wound Healing Potential of Photosensitive GelMA Hydrogel Incorporating Antimicrobial Peptides and MXene Nanoparticles. Front. Bioeng. Biotechnol..

[78] Atefyekta S., Blomstrand E., Rajasekharan A.K., Svensson S., Trobos M., Hong J., Webster T.J., Thomsen P., Andersson M. (2021). Antimicrobial Peptide-Functionalized Mesoporous Hydrogels. ACS Biomater. Sci. Eng..

[79] Chen Y., Qian H., Peng D., Jiang Y., Liu Q., Tan Y., Feng L., Cheng B., Li G. (2024). Antimicrobial Peptide-Modified AIE Visual Composite Wound Dressing for Promoting Rapid Healing of Infected Wounds. Front. Bioeng. Biotechnol..

[80] Martin-Serrano Á., Gómez R., Ortega P., de la Mata F.J. (2019). Nanosystems as Vehicles for the Delivery of Antimicrobial Peptides (Amps). Pharmaceutics.

[81] Campos J.V., de Pontes J.T.C., Canales C.S.C., Roque-Borda C.A., Pavan F.R. (2025). Advancing Nanotechnology: Targeting Biofilm-Forming Bacteria with Antimicrobial Peptides. BME Front..

[82] de Oliveira K.B.S., Leite M.L., Melo N.T.M., Lima L.F., Barbosa T.C.Q., Carmo N.L., Melo D.A.B., Paes H.C., Franco O.L. (2024). Antimicrobial Peptide Delivery Systems as Promising Tools Against Resistant Bacterial Infections. Antibiotics.

[83] Javia A., Misra A., Thakkar H. (2022). Liposomes Encapsulating Novel Antimicrobial Peptide Omiganan: Characterization and Its Pharmacodynamic Evaluation in Atopic Dermatitis and Psoriasis Mice Model. Int. J. Pharm..

[84] Changsan N., Atipairin A., Sakdiset P., Muenraya P., Balekar N., Srichana T., Sritharadol R., Phanapithakkun S., Sawatdee S. (2024). BrSPR-20-P1 Peptide Isolated from *Brevibacillus* sp. Developed into Liposomal Hydrogel as a Potential Topical Antimicrobial Agent. RSC Adv..

[85] Gelen-Gungor D., Nigiz Ş., Özkul C., Eroğlu H., Nemutlu E., Ulubayram K., Mao Y., Michniak-Kohn B., Eroğlu İ. (2025). Co-Delivery of Azithromycin and Nisin through Liposomes for Skin Infection to Reduce Antimicrobial Drug Resistance. Int. J. Pharm..

[86] Üstün A., Örtücü S. (2022). Evaluation of Nisin-Loaded PLGA Nanoparticles Prepared with Rhamnolipid Cosurfactant against *S. aureus* Biofilms. Pharmaceutics.

[87] Garcia-Orue I., Gainza G., Girbau C., Alonso R., Aguirre J.J., Pedraz J.L., Igartua M., Hernandez R.M. (2016). LL37 Loaded Nanostructured Lipid Carriers (NLC): A New Strategy for the Topical Treatment of Chronic Wounds. Eur. J. Pharm. Biopharm..

[88] Lin Q., Deslouches B., Montelaro R.C., Di Y.P. (2018). Prevention of ESKAPE Pathogen Biofilm Formation by Antimicrobial Peptides WLBU2 and LL37. Int. J. Antimicrob. Agents.

[89] Fumakia M., Ho E.A. (2016). Nanoparticles Encapsulated with LL37 and Serpin A1 Promotes Wound Healing and Synergistically Enhances Antibacterial Activity. Mol. Pharm..

[90] Rashki S., Safardoust-Hojaghan H., Mirzaei H., Abdulsahib W.K., Mahdi M.A., Salavati-Niasari M., Khaledi A., Khorshidi A., Mousavi S.G.A. (2022). Delivery LL37 by Chitosan Nanoparticles for Enhanced Antibacterial and Antibiofilm Efficacy. Carbohydr. Polym..

[91] Zhang Y., Håkansson J., Fan Y., Andrén O.C.J., San Jacinto García J., Qin L., Umerska A., Hutchinson D.J., Lüchow M., Mahlapuu M. (2023). Dendritic Nanogels Directed Dual-Encapsulation Topical Delivery System of Antimicrobial Peptides Targeting Skin Infections. Macromol. Biosci..

[92] Obuobi S., Mayandi V., Nor N.A.M., Lee B.J., Lakshminarayanan R., Ee P.L.R. (2020). Nucleic Acid Peptide Nanogels for the Treatment of Bacterial Keratitis. Nanoscale.

[93] Kumar M., Kumar D., Kumar D., Garg Y., Chopra S., Bhatia A. (2024). Therapeutic Potential of Nanocarrier Mediated Delivery of Peptides for Wound Healing: Current Status, Challenges and Future Prospective. AAPS PharmSciTech.

[94] Cesaro A., Lin S., Pardi N., de la Fuente-Nunez C. (2023). Advanced Delivery Systems for Peptide Antibiotics. Adv. Drug Deliv. Rev..

[95] Ren R., Lim C., Li S., Wang Y., Song J., Lin T.W., Muir B.W., Hsu H.Y., Shen H.H. (2022). Recent Advances in the Development of Lipid-, Metal-, Carbon-, and Polymer-Based Nanomaterials for Antibacterial Applications. Nanomaterials.

[96] Boge L., Hallstensson K., Ringstad L., Johansson J., Andersson T., Davoudi M., Larsson P.T., Mahlapuu M., Håkansson J., Andersson M. (2019). Cubosomes for Topical Delivery of the Antimicrobial Peptide LL-37. Eur. J. Pharm. Biopharm..

[97] Monfared Y.K., Mahmoudian M., Hoti G., Caldera F., Nicolás J.M.L., Zakeri-Milani P., Matencio A., Trotta F. (2022). Cyclodextrin-Based Nanosponges as Perse Antimicrobial Agents Increase the Activity of Natural Antimicrobial Peptide Nisin. Pharmaceutics.

[98] Carnero Canales C.S., Roque-Borda C.A., Negri A.C.C., Delgado O.R., Catarin Nunes L.O., Resende F.A., Barud H., Santos-Filho N.A., Garrido S.S., Oliveira K.C. (2026). Dual-Peptide Nanoplatform: Mesoporous Silica Nanoparticles Functionalized With a Cell-Penetrating Peptide and Loaded With Rationally Designed Antimicrobial Peptides for Tuberculosis Therapy. Adv. Healthc. Mater..

[99] Pang K., Wang W., Qin J.X., Shi Z.D., Hao L., Ma Y.Y., Xu H., Wu Z.X., Pan D., Chen Z.S. (2022). Role of Protein Phosphorylation in Cell Signaling, Disease, and the Intervention Therapy. MedComm.

[100] Wang H., Zhang X., Wang D., Jiang Q., Sun Y., Zhao B., Liang Z., Qing G., Jiang B., Zhang L. (2025). Affinity Peptide Ligands: New Tools for Chasing Non-Canonical N-Phosphoproteome. Chem. Sci..

[101] Leijten N.M., Heck A.J.R., Lemeer S. (2022). Histidine Phosphorylation in Human Cells; a Needle or Phantom in the Haystack?. Nat. Methods.

[102] Barbour A., Smith L., Oveisi M., Williams M., Huang R.C., Marks C., Fine N., Sun C., Younesi F., Zargaran S. (2023). Discovery of Phosphorylated Lantibiotics with Proimmune Activity That Regulate the Oral Microbiome. Proc. Natl. Acad. Sci. USA.

[103] Lin B., Qing X., Liao J., Zhuo K. (2020). Role of Protein Glycosylation in Host-Pathogen Interaction. Cells.

[104] Bellavita R., Braccia S., Galdiero S., Falanga A. (2023). Glycosylation and Lipidation Strategies: Approaches for Improving Antimicrobial Peptide Efficacy. Pharmaceuticals.

[105] Tortorella A., Leone L., Lombardi A., Pizzo E., Bosso A., Winter R., Petraccone L., Del Vecchio P., Oliva R. (2023). The Impact of N-Glycosylation on the Properties of the Antimicrobial Peptide LL-III. Sci. Rep..

[106] He M., Zhou X., Wang X. (2024). Glycosylation: Mechanisms, Biological Functions and Clinical Implications. Signal Transduct. Target. Ther..

[107] Bednarska N.G., Wren B.W., Willcocks S.J. (2017). The Importance of the Glycosylation of Antimicrobial Peptides: Natural and Synthetic Approaches. Drug Discov. Today.

[108] Maky M.A., Ishibashi N., Zendo T., Perez R.H., Doud J.R., Karmi M., Sonomoto K. (2015). Enterocin F4-9, a Novel O-Linked Glycosylated Bacteriocin. Appl. Environ. Microbiol..

[109] Maky M.A., Ishibashi N., Nakayama J., Zendo T. (2021). Characterization of the Biosynthetic Gene Cluster of Enterocin F4-9, a Glycosylated Bacteriocin. Microorganisms.

[110] Amso Z., Bisset S.W., Yang S.H., Harris P.W.R., Wright T.H., Navo C.D., Patchett M.L., Norris G.E., Brimble M.A. (2018). Total Chemical Synthesis of Glycocin F and Analogues: S-Glycosylation Confers Improved Antimicrobial Activity. Chem. Sci..

[111] Junior E.F.C., Guimarães C.F.R.C., Franco L.L., Alves R.J., Kato K.C., Martins H.R., de Souza Filho J.D., Bemquerer M.P., Munhoz V.H.O., Resende J.M. (2017). Glycotriazole-Peptides Derived from the Peptide HSP1: Synergistic Effect of Triazole and Saccharide Rings on the Antifungal Activity. Amino Acids.

[112] Ree R., Varland S., Arnesen T. (2018). Spotlight on Protein N-Terminal Acetylation. Exp. Mol. Med..

[113] Drazic A., Myklebust L.M., Ree R., Arnesen T. (2016). The World of Protein Acetylation. Biochim. Biophys. Acta Proteins Proteom..

[114] Alvares D.S., Wilke N., Ruggiero Neto J. (2018). Effect of N-Terminal Acetylation on Lytic Activity and Lipid-Packing Perturbation Induced in Model Membranes by a Mastoparan-like Peptide. Biochim. Biophys. Acta Biomembr..

[115] Van Neer R.H.P., Dranchak P.K., Liu L., Aitha M., Queme B., Kimura H., Katoh T., Battaile K.P., Lovell S., Inglese J. (2022). Serum-Stable and Selective Backbone-N-Methylated Cyclic Peptides That Inhibit Prokaryotic Glycolytic Mutases. ACS Chem. Biol..

[116] Li Y., Bionda N., Yongye A., Geer P., Stawikowski M., Cudic P., Martinez K., Houghten R.A. (2013). Dissociation of Antimicrobial and Hemolytic Activities of Gramicidin S through N-Methylation Modification. ChemMedChem.

[117] Zorzi A., Deyle K., Heinis C. (2017). Cyclic Peptide Therapeutics: Past, Present and Future. Curr. Opin. Chem. Biol..

[118] Hirano M., Saito C., Yokoo H., Goto C., Kawano R., Misawa T., Demizu Y. (2021). Development of Antimicrobial Stapled Peptides Based on Magainin 2 Sequence. Molecules.

[119] Mitra S., Chen M.T., Stedman F., Hernandez J., Kumble G., Kang X., Zhang C., Tang G., Reed I., Daugherty I.Q. (2025). Cyclization of Two Antimicrobial Peptides Improves Their Activity. ACS Omega.

[120] Ba Z., Wang Y., Yang Y., Ren B., Li B., Ouyang X., Zhang J., Yang T., Liu Y., Zhao Y. (2024). Phosphorylation as an Effective Tool to Improve Stability and Reduce Toxicity of Antimicrobial Peptides. J. Med. Chem..

[121] Dwivedi R., Aggarwal P., Bhavesh N.S., Kaur K.J. (2019). Design of Therapeutically Improved Analogue of the Antimicrobial Peptide, Indolicidin, Using a Glycosylation Strategy. Amino Acids.

[122] Cao R., Li L., Xu Z., Li J., Wu D., Wang Y., Zhu H. (2023). The Lipidation and Glycosylation Enabling Bioactivity Enhancement and Structural Change of Antibacterial Peptide G3. Bioorg. Med. Chem. Lett..

[123] Li D., Yang Y., Li R., Huang L., Wang Z., Deng Q., Dong S. (2021). N-Terminal Acetylation of Antimicrobial Peptide L163 Improves Its Stability against Protease Degradation. J. Pept. Sci..

[124] Humpola M.V., Spinelli R., Erben M., Perdomo V., Tonarelli G.G., Albericio F., Siano A.S. (2023). D- and N-Methyl Amino Acids for Modulating the Therapeutic Properties of Antimicrobial Peptides and Lipopeptides. Antibiotics.

[125] Rezende S.B., Oshiro K.G.N., Júnior N.G.O., Franco O.L., Cardoso M.H. (2021). Advances on Chemically Modified Antimicrobial Peptides for Generating Peptide Antibiotics. Chem. Commun..

[126] Van Gent M.E., Schonkeren-Ravensbergen B., Achkif A., Beentjes D., Dolezal N., Van Meijgaarden K.E., Drijfhout J.W., Nibbering P.H. (2023). C-Terminal PEGylation Improves SAAP-148 Peptide’s Immunomodulatory Activities. J. Innate Immun..

[127] Kamysz E., Sikorska E., Jaśkiewicz M., Bauer M., Neubauer D., Bartoszewska S., Barańska-Rybak W., Kamysz W. (2020). Lipidated Analogs of the Ll-37-Derived Peptide Fragment KR12—Structural Analysis, Surface-Active Properties and Antimicrobial Activity. Int. J. Mol. Sci..

[128] Peng X., Luo Y., Xu T., Chen Z., Chen P., Hu C., Liu S. (2025). Antibiotic-Conjugated Antimicrobial Peptides for Enhanced Bacterial Inhibition. RSC Adv..

[129] Ramirez D., Berry L., Domalaon R., Brizuela M., Schweizer F. (2020). Dilipid Ultrashort Tetrabasic Peptidomimetics Potentiate Novobiocin and Rifampicin against Multidrug-Resistant Gram-Negative Bacteria. ACS Infect. Dis..

[130] Jelinkova P., Splichal Z., Jimenez A.M.J., Haddad Y., Mazumdar A., Sur V.P., Milosavljevic V., Kopel P., Buchtelova H., Guran R. (2018). Novel Vancomycin–Peptide Conjugate as Potent Antibacterial Agent against Vancomycin-Resistant *Staphylococcus aureus*. Infect. Drug Resist..

[131] Khazaal M.T., Faraag A.H.I., El-Hendawy H.H. (2024). In Vitro and in Silico Studies of Enterobactin-Inspired Ciprofloxacin and Fosfomycin First Generation Conjugates on the Antibiotic Resistant *E. coli* OQ866153. BMC Microbiol..

[132] Rohrbacher C., Zscherp R., Weck S.C., Klahn P., Ducho C. (2023). Synthesis of an Antimicrobial Enterobactin-Muraymycin Conjugate for Improved Activity Against Gram-Negative Bacteria. Chem.—A Eur. J..

[133] Olshvang E., Fritsch S., Scholtyssek O.C., Schalk I.J., Metzler-Nolte N. (2023). Vectorization via Siderophores Increases Antibacterial Activity of K(RW)3 Peptides against *Pseudomonas aeruginosa*. Chem.—A Eur. J..

[134] Roque-Borda C.A., Zhang Q., de la Torre B.G., Albericio F., Perdigão J., Pavan F.R. (2026). From Antibiotic to Peptide Siderophore Conjugates as Modular Strategies against Multidrug-Resistant Bacteria. Clin. Microbiol. Rev..

[135] Li T., Yang N., Teng D., Mao R., Hao Y., Wang X., Wang J. (2022). C-Terminal Mini-PEGylation of a Marine Peptide N6 Had Potent Antibacterial and Anti-Inflammatory Properties against *Escherichia coli* and *Salmonella* Strains in Vitro and in Vivo. BMC Microbiol..

[136] Roscetto E., Bellavita R., Paolillo R., Merlino F., Molfetta N., Grieco P., Buommino E., Catania M.R. (2021). Antimicrobial Activity of a Lipidated Temporin l Analogue against Carbapenemase-Producing *Klebsiella pneumoniae* Clinical Isolates. Antibiotics.

[137] Van Groesen E., Slingerland C.J., Innocenti P., Mihajlovic M., Masereeuw R., Martin N.I. (2021). Vancomyxins: Vancomycin-Polymyxin Nonapeptide Conjugates That Retain Anti-Gram-Positive Activity with Enhanced Potency against Gram-Negative Strains. ACS Infect. Dis..

[138] Bellavita R., Braccia S., Imbò L.E., Grieco P., Galdiero S., D’Auria G., Falanga A., Falcigno L. (2024). Exploring Fe(III) Coordination and Membrane Interaction of a Siderophore-Peptide Conjugate: Enhancing Synergistically the Antimicrobial Activity. J. Inorg. Biochem..

[139] Thomas X., Destoumieux-Garzón D., Peduzzi J., Afonso C., Blond A., Birlirakis N., Goulard C., Dubost L., Thai R., Tabet J.C. (2004). Siderophore Peptide, a New Type of Post-Translationally Modified Antibacterial Peptide with Potent Activity. J. Biol. Chem..

[140] Johnson K., Delaney J.C., Guillard T., Reffuveille F., Varin-Simon J., Li K., Wollacott A., Frapy E., Mong S., Tissire H. (2023). Development of an Antibody Fused with an Antimicrobial Peptide Targeting *Pseudomonas aeruginosa*: A New Approach to Prevent and Treat Bacterial Infections. PLoS Pathog..

[141] Touti F., Lautrette G., Johnson K.D., Delaney J.C., Wollacott A., Tissire H., Viswanathan K., Shriver Z., Mong S.K., Mijalis A.J. (2018). Antibody–Bactericidal Macrocyclic Peptide Conjugates To Target Gram-Negative Bacteria. ChemBioChem.

[142] Ajayi O.E., Bellavita R., Imbò L.E., Palladino S., Braccia S., Falanga A., Galdiero S. (2025). Boosting AMPs’ Power: From Structural Engineering to Nanotechnology-Based Delivery. Molecules.

[143] Gagat P., Ostrówka M., Duda-Madej A., Mackiewicz P. (2024). Enhancing Antimicrobial Peptide Activity through Modifications of Charge, Hydrophobicity, and Structure. Int. J. Mol. Sci..

[144] Yang R., Ma X., Peng F., Wen J., Allahou L.W., Williams G.R., Knowles J.C., Poma A. (2025). Advances in Antimicrobial Peptides: From Mechanistic Insights to Chemical Modifications. Biotechnol. Adv..

[145] Mwangi J., Kamau P., Thuku R., Lai R. (2023). Design Methods for Antimicrobial Peptides with Improved Performance. Zool. Res..

[146] Liu L., Ni D., Yan Y., Wu S., Chen X., Guan J., Xiong X., Liu G. (2020). Development of a Novel DNA Delivery System Based on Rice Bran Polysaccharide-Fe(III) Complexes. Int. J. Biol. Macromol..

[147] Roberts K.D., Zhu Y., Azad M.A.K., Han M.-L., Wang J., Wang L., Yu H.H., Horne A.S., Pinson J.-A., Rudd D. (2022). A Synthetic Lipopeptide Targeting Top-Priority Multidrug-Resistant Gram-Negative Pathogens. Nat. Commun..

[148] Jung Kim D., Lee Y.W., Park M.K., Shin J.R., Lim K.J., Cho J.H., Kim S.C. (2014). Efficacy of the Designer Antimicrobial Peptide SHAP1 in Wound Healing and Wound Infection. Amino Acids.

[149] Håkansson J., Ringstad L., Umerska A., Johansson J., Andersson T., Boge L., Rozenbaum R.T., Sharma P.K., Tollbäck P., Björn C. (2019). Characterization of the in Vitro, Ex Vivo, and in Vivo Efficacy of the Antimicrobial Peptide DPK-060 Used for Topical Treatment. Front. Cell. Infect. Microbiol..

[150] Gomes A., Leal E.C., Da Silva J., Teixeira I., Ferraz R., Calheiros D., Gonçalves T., Carvalho E., Gomes P. (2025). Enhancement of Wound Healing in Diabetic Mice by Topical Use of a Peptide-Ionic Liquid Conjugate. Int. J. Biochem. Cell Biol..

[151] Kang M., Hwang W., Yang J., Kim S., Jeon S., Noh M., Lee J., Lee J.B., Kim Y., Kim J.W. (2025). Cell-Penetrating Peptide-Engineered Solid Lipid Nanoparticles for Enhanced Endocytotic Internalization and Transdermal Penetration. ACS Appl. Nano Mater..

[152] Chen P., Sebastian E.A., Karna S.L.R., Leung K.P. (2024). Development of a Stringent Ex Vivo-Burned Porcine Skin Wound Model to Screen Topical Antimicrobial Agents. Antibiotics.

[153] Alencar-Silva T., Díaz-Martín R.D., Sousa dos Santos M., Saraiva R.V.P., Leite M.L., de Oliveira Rodrigues M.T., Pogue R., Andrade R., Falconi Costa F., Brito N. (2024). Screening of the Skin-Regenerative Potential of Antimicrobial Peptides: Clavanin A, Clavanin-MO, and Mastoparan-MO. Int. J. Mol. Sci..

[154] Ross A., Guo X., Salazar G.A.M., Schejtman S.D.G., El-Hage J., Comtois-Bona M., Macadam A., Guzman-Soto I., Takaya H., Hu K. (2024). Multipurpose On-the-Spot Peptide-Based Hydrogels for Skin, Cornea, and Heart Repair. Adv. Funct. Mater..

[155] Andersson M., Madsen L.B., Schmidtchen A., Puthia M. (2021). Development of an Experimental Ex Vivo Wound Model to Evaluate Antimicrobial Efficacy of Topical Formulations. Int. J. Mol. Sci..

[156] Lima W.G., de Lima M.E. (2023). Therapeutic Prospection of Animal Venoms-Derived Antimicrobial Peptides against Infections by Multidrug-Resistant *Acinetobacter baumannii*: A Systematic Review of Pre-Clinical Studies. Toxins.

[157] Takahashi M., Umehara Y., Yue H., Trujillo-Paez J.V., Peng G., Nguyen H.L.T., Ikutama R., Okumura K., Ogawa H., Ikeda S. (2021). The Antimicrobial Peptide Human β-Defensin-3 Accelerates Wound Healing by Promoting Angiogenesis, Cell Migration, and Proliferation Through the FGFR/JAK2/STAT3 Signaling Pathway. Front. Immunol..

[158] Zou K., Zhang S., Yin K., Ren S., Zhang M., Li X., Fan L., Zhang R., Li R. (2024). Studies on the in Vitro Mechanism and in Vivo Therapeutic Effect of the Antimicrobial Peptide ACP5 against Trichophyton Mentagrophytes. Peptides.

[159] Heunis T.D.J., Smith C., Dicks L.M.T. (2013). Evaluation of a Nisin-Eluting Nanofiber Scaffold to Treat *Staphylococcus aureus*-Induced Skin Infections in Mice. Antimicrob. Agents Chemother..

[160] Xiong Y.Q., Li L., Zhou Y., Kraus C.N. (2019). Efficacy of ARV-1502, a Proline-Rich Antimicrobial Peptide, in a Murine Model of Bacteremia Caused by Multi-Drug Resistant (MDR) Acinetobacter Baumannii. Molecules.

[161] van Gent M.E., Ali M., Nibbering P.H., Kłodzińska S.N. (2021). Current Advances in Lipid and Polymeric Antimicrobial Peptide Delivery Systems and Coatings for the Prevention and Treatment of Bacterial Infections. Pharmaceutics.

